# Ubiquitylome analysis reveals the protective effect of α-lipoic acid via the ferroptosis pathway during recovery after hepatic oxidative stress in finishing pigs

**DOI:** 10.1007/s44154-026-00341-1

**Published:** 2026-09-23

**Authors:** Jie Gao, Yanjun Cui, Chenyang Li, Weiguang Bao, Yue Hao, Xiangshu Piao, Qingshi Meng, Xianhong Gu

**Affiliations:** 1 State Key Laboratory of Animal Nutrition and Feeding, Institute of Animal Science, Chinese Academy of Agricultural Sciences, Beijing, 100193 China; 2 State Key Laboratory of Animal Nutrition and Feeding, College of Animal Science and Technology, China Agricultural University, Beijing, 100193 China

**Keywords:** Oxidative Stress, Ubiquitination, α-Lipoic Acid, Liver, Finishing Pigs

## Abstract

Ubiquitination is a critical post-translational modification that regulates hepatic antioxidant defense. α-Lipoic acid (LA) exerts potent hepatoprotective effects, but its mechanism of regulating ubiquitination in porcine livers with oxidative injury, especially during the recovery period, remains unclear. In this study, 18 castrated male Large White (LW) pigs were randomly assigned to control, diquat (DQ), and diquat + LA (DL, 800 mg/kg diet) groups. On day 15, the DQ and DL groups were injected with diquat (8 mg/kg body weight), and liver tissues were sampled on day 29. Tandem mass tag (TMT)-based proteomic analysis quantified 5,326 of 7,271 identified proteins, which were validated by parallel reaction monitoring (PRM). Label-free ubiquitylome analysis identified 1,651 ubiquitination sites corresponding to 1,446 peptides and 594 ubiquitinated proteins, among which 1,208 sites, 1,058 peptides, and 489 proteins were quantified. Our findings revealed that LA upregulated ferritin heavy chain 1 (FTH1) expression and promoted Poly(rC)-binding protein 1 (PCBP1) ubiquitination at K314 in porcine liver during recovery after hepatic oxidative stress; these two proteins are core regulators of iron storage in ferroptosis pathway. Hepatic antioxidant status and histopathological analysis confirmed that dietary LA effectively balanced the compensatory activation of antioxidant system and alleviated oxidative stress-induced liver injury. Cell-based assays further verified that LA significantly ameliorated the reduction in FTH1 and the elevation in Fe^2+^ concentration induced by oxidative stress in a concentration-dependent manner. Collectively, we propose that LA protects hepatocytes by enhancing iron sequestration, inhibiting lipid peroxidation, and suppressing ferroptosis. This study provides novel insights into how LA maintains hepatic redox homeostasis by regulating the expression and ubiquitination of key proteins in the ferroptosis pathway.

**Supplementary Information:**

The online version contains supplementary material available at https://doi.org/10.1007/s44154-026-00341-1.

## Introduction

The liver, as a core metabolic organ in pigs, is involved in numerous physiological processes such as nutrient metabolism, toxin degradation, and immune regulation, making it highly susceptible to oxidative stress-induced damage (Mooli et al. 2023; Hwang et al. 2025). In modern intensive farming systems, factors including early weaning, dietary imbalance, and environmental stressors can disrupt the redox homeostasis in porcine liver, leading to excessive accumulation of reactive oxygen species (ROS) (Allameh et al. 2023; Hong et al. 2024; Yu et al. 2024; Golubska et al. 2025; Wu et al. 2025). Oxidative stress not only impairs hepatic function and reduces growth performance but also poses a significant threat to the health and welfare of pigs, ultimately causing substantial economic losses to the swine industry (Yin et al. 2024). Therefore, elucidating the molecular mechanisms underlying the antioxidant defense system in porcine liver and identifying effective regulatory strategies have become critical research priorities in porcine nutrition and health.

In the liver, iron, as an essential trace element, participates in physiological processes such as hemoglobin synthesis and electron transport (Gensluckner et al. 2024). Ferroptosis is an iron-dependent form of programmed cell death, characterized by massive accumulation of lipid peroxides and disrupted iron homeostasis, which is widely involved in the occurrence and development of liver diseases (Wu et al. 2021; Hino et al. 2025). In models such as metabolic-associated steatohepatitis and acute liver ischemia–reperfusion injury, hepatocyte ferroptosis serves as a key link exacerbating liver injury. In the porcine liver, oxidative stress-induced ferroptosis has also been proven to be tightly associated with liver injury, alongside the abnormal activation of its core regulatory pathways (Zhang et al. 2024). However, the upstream regulatory mechanisms of ferroptosis in the porcine liver, especially the roles of post-translational modifications in regulating the ferroptosis pathway, remain to be further clarified.

Ubiquitination, as a key post-translational modification, regulates the degradation, localization, and activity of target proteins through the E1-E2-E3 ubiquitin ligase cascade reaction, and plays a core role in cellular stress response, iron homeostasis regulation, and ferroptosis process (Meng et al. 2022; Wu et al. 2022; Din et al. 2024). Accumulating evidence indicates that ubiquitin system enzymes or their complexes regulate the susceptibility of cells to ferroptosis, mainly by regulating ubiquitination and the protein stability of key regulators of ferroptosis (Ding et al. 2021; Wang et al. 2021, 2023; Chen et al. 2022a, b). Notably, the stability and function of multiple key regulatory proteins in the ferroptosis pathway are regulated by ubiquitination. Specific ubiquitin ligases can mediate the ubiquitination and degradation of these relevant proteins, thereby participating in tissue damage induced by iron homeostasis imbalance. Recent studies have shown that ubiquitination can regulate the ferroptosis process by targeting key proteins in the ferroptosis pathway; for example, the E3 ubiquitin ligase Parkin can inhibit myocardial cell ferroptosis by ubiquitinating and degrading the key ferroptosis regulator acyl-CoA synthetase long-chain family member 4 (ACSL4) (Xiao et al. 2025); while the E3 ubiquitin ligase HUWE1 inhibits ferroptosis in acute liver injury by ubiquitinating and degrading the transferrin receptor 1 (TfR1), thereby diminishing cellular iron influx (Wu et al. 2022). Other studies have shown that the deubiquitinating enzyme OTUB1 is recruited under the effect of removing the ubiquitin chain on solute carrier family 7 member 11 (SLC7A11) to prevent its degradation through the proteasome and improve its stability, thereby inhibiting the occurrence of ferroptosis in tumors (Liu et al. 2019). Glutathione peroxidase 4 (GPX4), a key ferroptosis regulator that antagonizes cellular lipid peroxidation, undergoes multiple ubiquitination modifications (Bersuker et al. 2019; Dong et al. 2022). Collectively, these studies indicate that ubiquitination may act as a core regulatory hub connecting oxidative stress and ferroptosis by modulating key proteins in the ferroptosis pathway. However, the ubiquitination-modulated proteins participating in the ferroptosis pathway of oxidatively injured porcine liver (especially during the recovery period), together with the molecular mechanisms by which ubiquitination mediates hepatic adaptive responses after oxidative injury, remain to be further elaborated.α-Lipoic acid (LA) is a natural dithiol compound with both lipophilic and hydrophilic properties, known as a "universal antioxidant", which is widely present in metabolically active tissues such as the liver and heart (Superti and Russo 2024). LA and its reduced form dihydrolipoic acid (DHLA) alleviate oxidative damage through multiple pathways, such as directly scavenging ROS, chelating transition metal ions such as Fe^2+^, and regenerating endogenous antioxidants such as glutathione (Liu et al. 2020). Previous studies have confirmed that LA can improve tissue damage in various disease models such as diabetic nephropathy and myocardial ischemia–reperfusion injury by inhibiting ferroptosis (Li et al. 2021), but whether its mechanism of action is associated with the regulation of ubiquitination remains unclear. Considering the metal chelating properties and antioxidant potential of LA, as well as the core role of ubiquitination in ferroptosis regulation, we speculate that LA may target key proteins in the ferroptosis pathway by regulating ubiquitination, thereby improving the antioxidant function of porcine liver.

To explore the mechanism by which LA modulates hepatic antioxidant capacity through regulating the abundance and ubiquitination of core functional proteins in livers with oxidative injury, especially during the recovery period, this study analyzed the differentially expressed proteins (DEPs) among different groups in the livers of oxidatively stressed pigs using tandem mass tag (TMT)-based liquid chromatography-tandem mass spectrometry (LC–MS/MS). Parallel reaction monitoring (PRM) technology was further adopted to examine expression changes of candidate DEPs and verify the reliability of TMT-based protein quantification results. Qualitative and quantitative information on differentially ubiquitinated proteins (DUPs) and corresponding ubiquitination sites in porcine liver was obtained by analyzing ubiquitinated proteins using LC–MS/MS-based label-free protein quantification technology. Combined with assessments of hepatic antioxidant status and the expression of key proteins, we preliminarily explore the mechanism by which functional feed additives regulate the body's antioxidant capacity through the post-translational modifications (PTMs) pathway.

## Results

### Effects of LA on protein abundance and ubiquitination in porcine liver during recovery after oxidative stress

**Fig. 1:**
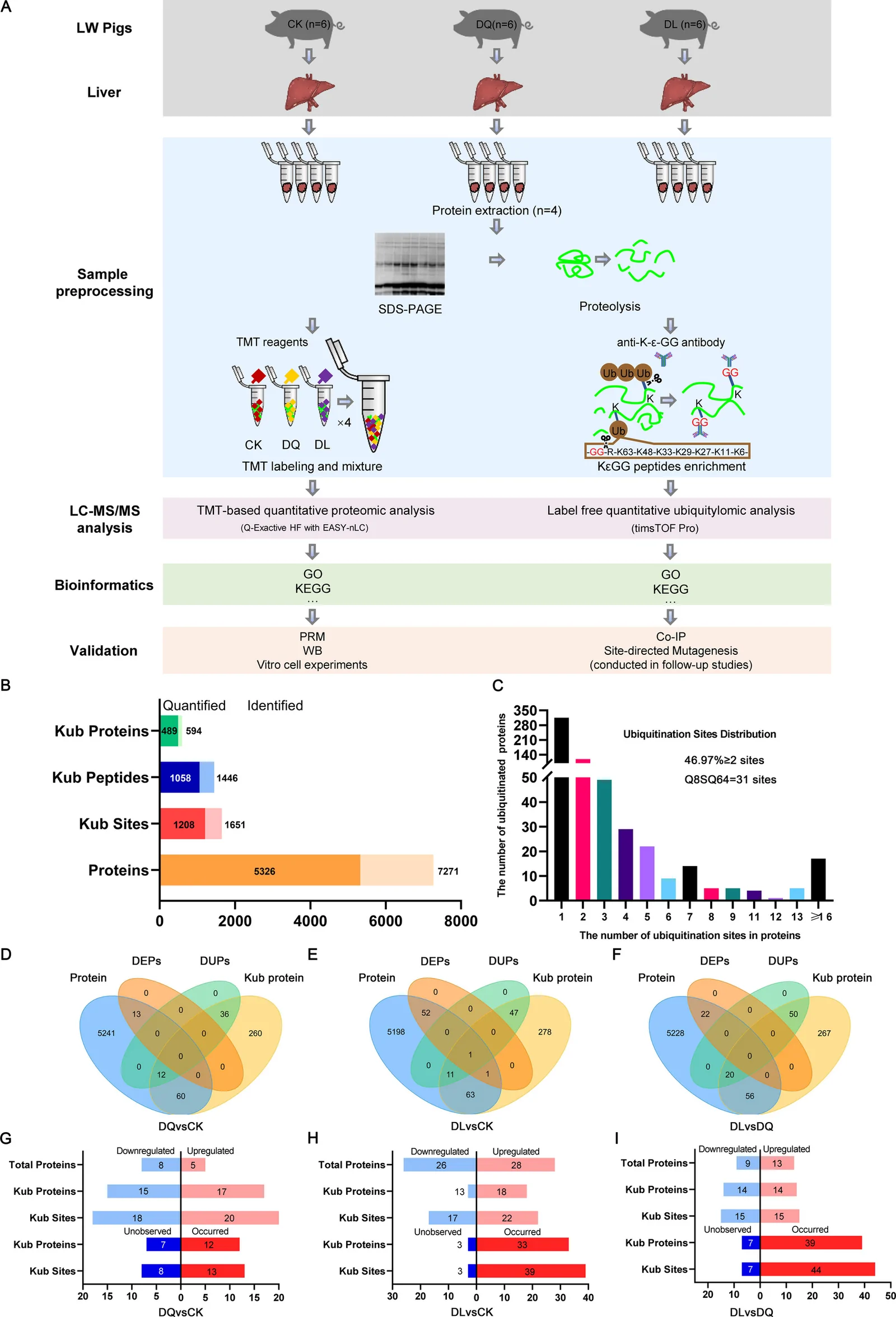
Overview of global proteome and ubiquitylome analysis in porcine liver (n = 4). **A** Animal grouping, proteome, and ubiquitylome analysis workflow; **B** Statistical overview of identification and quantification in proteomics and ubiquitinomics; **C** Distribution of Kub sites and statistics of corresponding proteins; **D-F** Pairwise Venn diagrams showing overlaps of proteins, Kub proteins, differentially expressed proteins (DEPs), and differentially ubiquitinated proteins (DUPs) across different groups; **G-I** Quantitative results of DEPs, DUPs, and differentially Kub sites among groups. CK, control group; DQ, diquat group; DL, diquat + LA group; LW pigs, Large White finishing pigs; Kub proteins, lysine-ubiquitinated proteins; Kub sites, lysine-ubiquitination sites. The proteins with FC (fold change) ≥ 1.2 or ≤ 0.83 and *p* < 0.05 were defined as DEPs; Kub sites with FC ≥ 2.0 or ≤ 0.5 and *p* < 0.05 were defined as differentially Kub sites, and their corresponding proteins are DUPs

To further clarify the regulatory function of LA on protein expression and ubiquitination in livers with oxidative injury, especially in the recovery period, TMT labeling combined with LC–MS/MS was applied to identify DEPs among CK, DQ and DL groups (Fig. 1A, Table 1), and PRM analysis was conducted to verify the expression changes of candidate DEPs. In this study, immunoaffinity enrichment and mass spectrometry analysis of ubiquitinated peptides from hepatic proteins of finishing pigs in each group were performed via label-free protein quantification technology (Fig. 1A, Table 2).

**Table 1:** Differentially expressed proteins related to hepatic antioxidant function during recovery after oxidative stress

Accession	Gene name	Protein name	Changes	Groups
P79383	CYP2E1	Cytochrome P450 2E1	↓	DQvsCK
A0A480PUC2	-	Peroxisomal multifunctional enzyme type 2 isoform 2	↓	DLvsCK
A0A4X1W9I5	*METTL7B*	Methyltransf_11 domain-containing protein	↓	DLvsCK
F1RIF8	*PGD*	2-phosphogluconate dehydrogenase, decarboxylating	↓	DLvsCK
P50441	*GATM*	Glycine amidinotransferase, mitochondrial	↑	DLvsCK
D0G6Y4	*HSD3B*	Hydroxy-delta-5-steroid dehydrogenase, 3 beta-and steroid delta-isomerase 1	↓	DLvsCK
A0A4X1SI12	*HSD17B12*	Uncharacterized protein	↓	DLvsCK
A0A287BQR3	*FCN2*	Ficolin-2	↓	DLvsCK
A0A287BQR3	*FCN2*	Ficolin-2	↓	DLvsDQ
A0A4X1V7C5	*FMO3*	Dimethylaniline monooxygenase [N-oxide-forming]	↓	DLvsCK
A0A4X1VGP9	*FTH1*	Ferritin	↑	DLvsCK
A0A4X1VYT5	-	Ferritin	↑	DLvsCK
A0A4X1VGP9	*FTH1*	Ferritin	↑	DLvsDQ
Q95314	-	Ferritin (Fragment)	↑	DLvsDQ
A0A4X1VYT5	-	Ferritin	↑	DLvsDQ

Note: FC = fold change, the fold change in protein expression between groups (n = 4); Proteins with FC ≥ 1.2 (↑, up-regulated) or ≤ 0.83 (↓, down-regulated) and *p* < 0.05 were screened as differentially expressed proteins

**Table 2:** Differential ubiquitination sites and ubiquitinated proteins related to hepatic antioxidant function during recovery after oxidative stress

Accession	Gene name	Kub sites	Groups	Changes
Q9MZS9	*KMO*	THLVK(+114.04)KPR	DLvsCK	detected
I3LEC2	*PCBP1*	QMSGAQIK(+114.04)IANPVEGSSGR	DLvsDQ	detected
A0A4X1U3G2	*ALDH1L1*	FAELTLK(+114.04)AGIPK	DLvsCK	detected
		LPQPEEGATYEGIQK(+114.04)K		detected
A0A286ZK24	*HPD*	T(+42.01)SYSDKGEK(+114.04)PER	DLvsCK	↑
		T(+42.01)SYSDK(+114.04)GEKPER		↑
		GAIIVREPWIEQDKFGK(+114.04)VK		detected
A0A287A8H8	*ALDH2*	TVTVK(+114.04)VPQK	DQvsCK	↓
A0A4X1SHI9	*MAT1A*	DTIK(+114.04)HIGYDDSAK	DQvsCK	↑
A0A4X1SSN8	*CAT*	IQALLDKYNAEK(+114.04)PK	DQvsCK	↓
		IQALLDKYNAEKPK(+114.04)		↓
		IQALLDKYNAEK(+114.04)PK(+114.04)		undetected
		QALLDK(+114.04)YNAEKPKNAVHTYVQAGSHLAAR	DLvsDQ	↓
		IQALLDKYNAEK(+114.04)PK(+114.04)		detected
A0A4X1W9I5	*METTL7B*	WLPVGPHIMGK(+114.04)AVK	DLvsCK	↓
		WLPVGPHIM(+15.99)GK(+114.04)AVK		↓
		LPVGPHIMGK(+114.04)AVK		↓
		KDLESAK(+114.04)FSELQM(+15.99)EQHPPLLK		detected
		KDLESAK(+114.04)FSELQMEQHPPLLK		detected
		LPVGPHIM(+15.99)GK(+114.04)AVK		undetected
		WLPVGPHIM(+15.99)GK(+114.04)AVK	DLvsDQ	↓
		KDLESAK(+114.04)FSELQM(+15.99)EQHPPLLK		detected
		KDLESAK(+114.04)FSELQMEQHPPLLK		detected
A0A4X1WBH4	*PRDX1*	DISLSDYK(+114.04)GK	DQvsCK	detected
		S(+42.01)SGNAK(+114.04)IGHR	DLvsDQ	↓
		DISLSDYK(+114.04)GK		undetected
A0A5G2QML3	*H1-3*	S(+42.01)ETAPVAPAAPAPAEKTPVK(+114.04)	DLvsCK	↑
		K(+114.04)ASGPPVSELITK		detected
A0A5G2RC42	*PYGL*	YEYGIFNQK(+114.04)IR	DLvsDQ	↑
A0A5S6G3Y8	*HSPB1*	AQLGGTEAGKSEKPGTK(+114.04)	DLvsCK	↑
		AQLGGTEAGK(+114.04)SEKPGTK		↑
		TKDGVVEITGK(+114.04)HEERQDEHGFISR		detected
		DGVVEITGK(+114.04)HEERQDEHGFISR	DLvsDQ	↑
		AQLGGTEAGK(+114.04)SEKPG		detected
F1SUN1	*SLCO2B1*	KVLAVSDSPVSKGEDSPSEQSPGASPEKK(+114.04)	DQvsCK	↑
		SPSEQSPGASPEK(+114.04)K		undetected
Q8SQ64	*CYP2E1*	YSDHFK(+114.04)AF	DQvsCK	↓
		IPAIK(+114.04)DR		detected

Note: Lysine residues annotated with K(+114.04) indicate the ubiquitination sites on the corresponding peptides. FC = fold change, the fold change in ubiquitination level between groups (n = 4); FC ≥ 2.0 (↑, up-regulated) or ≤ 0.50 (↓, down-regulated) and *p* < 0.05 were selected as differentially ubiquitinated sites. Kub sites = lysine-ubiquitination sites

By TMT combined with LC–MS/MS analysis, a total of 7271 proteins were identified, of which 5326 proteins were quantified; 594 ubiquitinated proteins, 1446 ubiquitinated peptides and 1651 ubiquitination sites were identified through ubiquitylome analysis in this study, of which 1058 ubiquitinated peptides and 1208 ubiquitination sites were quantifiable among 489 quantifiable ubiquitinated proteins (Fig. 1B; Additional file 1; Additional file 2). In order to analyze the distribution of ubiquitination sites on proteins, we identified the number of ubiquitination sites on all qualitatively identifiable proteins, and the results showed that 46.97% of proteins had more than two ubiquitination sites, among which Q8SQ64 protein contained up to 31 ubiquitination sites (Fig. 1C). Pairwise Venn diagrams inllustrated overlaps of proteins, Kub proteins, DEPs, and DUPs across different groups (Fig. 1D-F). In addition, among these quantified proteins, 13 (up-regulated: 5, down-regulated: 8) were differentially expressed between the DQ and CK groups, and 54 (up-regulated: 28, down-regulated: 26) were differentially expressed between the DL and CK groups, 21 (up-regulated: 13, down-regulated: 9) were differentially expressed between the DL and DQ groups (Fig. 1G-I). Compared with CK group, 20 (52.6%) ubiquitination sites of 17 ubiquitinated proteins were up-regulated and 18 (47.4%) ubiquitination sites of 15 ubiquitinated proteins were down-regulated in the livers of DQ group, which were intraperitoneally injected with diquat (Fig. 1G). Compared with CK group, 22 (56.4%) ubiquitination sites of 18 ubiquitinated proteins were up-regulated and 17 (43.6%) ubiquitination sites of 13 ubiquitinated proteins were down-regulated in liver of DL group (Fig. 1H). Compared with the DQ group, 15 (50.0%) ubiquitination sites of 14 ubiquitinated proteins were up-regulated and 15 (50.0%) ubiquitination sites of 14 ubiquitinated proteins were down-regulated in the livers of pigs in the DL group, which were fed a diet with 800 mg LA/kg diet and intraperitoneally injected with diquat (Fig. 1I). In addition, this study identified ubiquitination sites and corresponding proteins that exhibited presence/absence differences between every two groups of porcine livers (Table 2). Specifically, these ubiquitination modifications were detected in only one group but absent in the paired comparison group.

### Biological function analysis of DEPs and DUPs

**Fig. 2:**
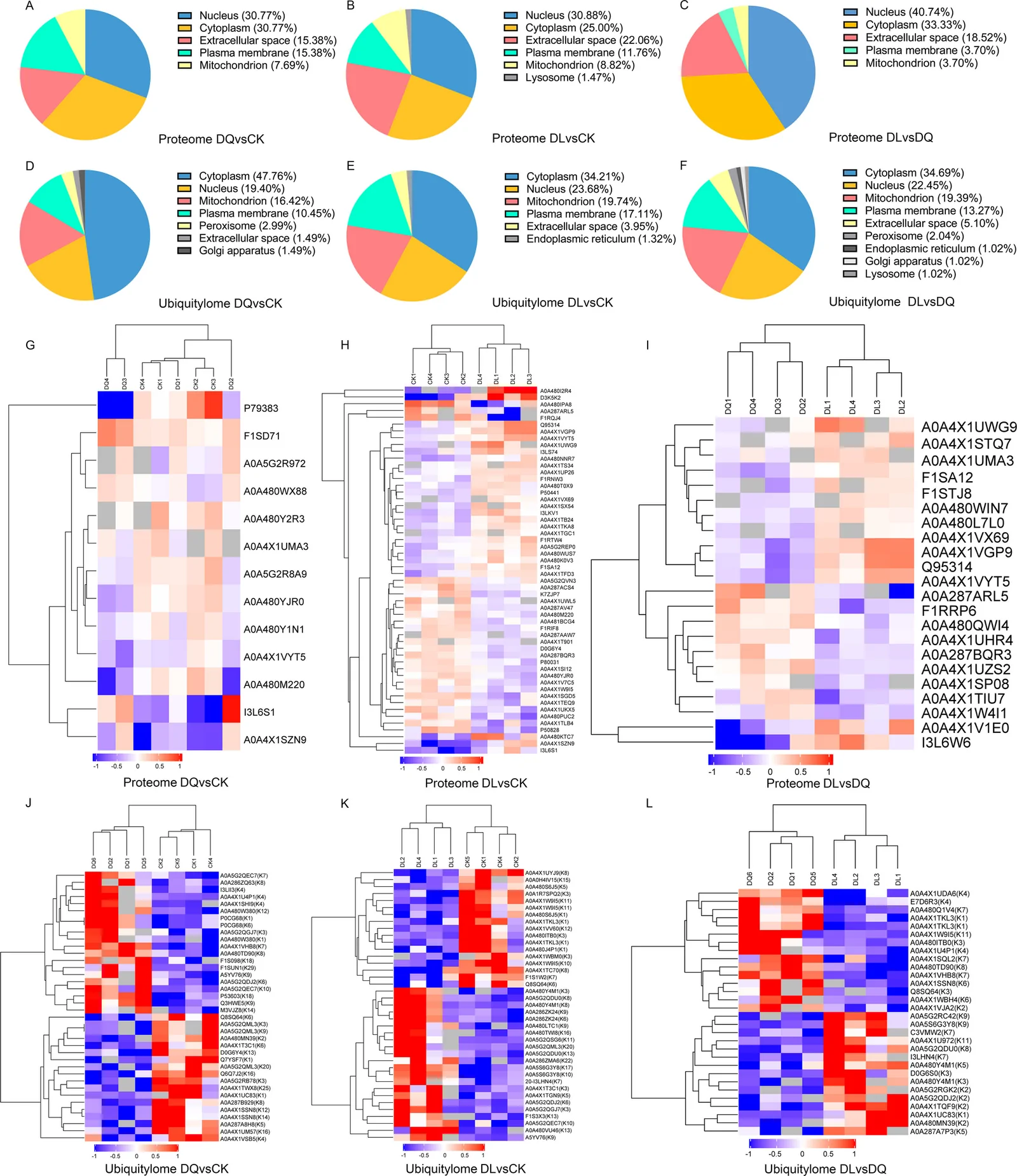
Subcellular classification and clustering analysis of differentially expressed proteins (DEPs) and differentially ubiquitinated proteins (DUPs) in porcine liver. Subcellular localization analysis of DEPs (**A-C**) and DUPs (**D-F**); clustering analysis of DEPs (**G-I**) and differentially ubiquitinated peptides (**J-L**), n = 4, each row represents a protein or a ubiquitinated peptide, and each column represents a biological replicate, red indicates up-regulated proteins/ubiquitinated peptides, blue indicates down-regulated proteins/ubiquitinated peptides, and gray indicates no quantitative information. CK, control group; DQ, diquat group; DL, diquat + LA group

To elucidate distinct hepatic cellular processes modulated by LA in the liver of oxidatively injured finishing pigs, especially during the recovery period, we analyzed the subcellular localization and protein domains of DEPs and DUPs identified in porcine liver. The results showed that DEPs between the DQ and CK groups were mainly localized in nucleus (30.77%) and cytoplasm (30.77%) (Fig. 2A); the DUPs were mainly located in the cytoplasm (47.76%) and nucleus (19.40%) (Fig. 2D). DEPs between the DL and CK groups were mainly localized in nucleus (30.88%) and cytoplasm (25.00%) (Fig. 2B); the DUPs were mainly located in the cytoplasm (34.21%) and nucleus (23.68%) (Fig. 2E). DEPs between the DL and DQ groups were mainly localized in nucleus (40.74%) and cytoplasm (33.33%) (Fig. 2C); the DUPs were mainly located in the cytoplasm (34.69%) and nucleus (22.45%) (Fig. 2F). Through inter-group clustering analysis of DEPs (Fig. 2G-I) and differentially ubiquitinated peptides (Fig. 2J-L), it was found that except for the low inter-group discrimination between the DQ and CK groups based on proteomic results (Fig. 2G), all other groups showed excellent intra-group parallelism and inter-group differentiation in general, reflecting that the screening of differentially expressed ubiquitinated peptides can represent the impact of biological treatments on samples.

The domain is the fundamental unit of protein structure and function, and the prediction of domains is significant for analyzing the potential biological roles of proteins. The results show that DEPs between the DQ and CK groups are primarily associated with metallothionein, ferritin-like domains, nucleotidyltransferase domain, and ubiquitin-conjugating enzyme, etc. (Additional file 3: Fig. S1 A), while DUPs are mainly related to cytochrome P450 (Additional file 3: Fig. S1 B). Between the DL and CK groups, DEPs are primarily associated with ferritin-like domains, metallothionein, and short-chain dehydrogenase, etc. (Additional file 3: Fig. S1 C), while DUPs are mainly related to flavin-binding monooxygenase-like, cytochrome P450, linker histone H1 and H5 family, and phosphopantetheine attachment site (Additional file 3: Fig. S1 D). Between the DL and DQ groups, DEPs are primarily associated with ferritin-like domains, immunoglobulin V-set domains, and linker histone H1 and H5 family, etc. (Additional file 3: Fig. S1 E), while DUPs are mainly related to cytochrome P450, ubiquitin carboxyl-terminal hydrolase, etc. (Additional file 3: Fig. S1 F).

**Fig. 3:**
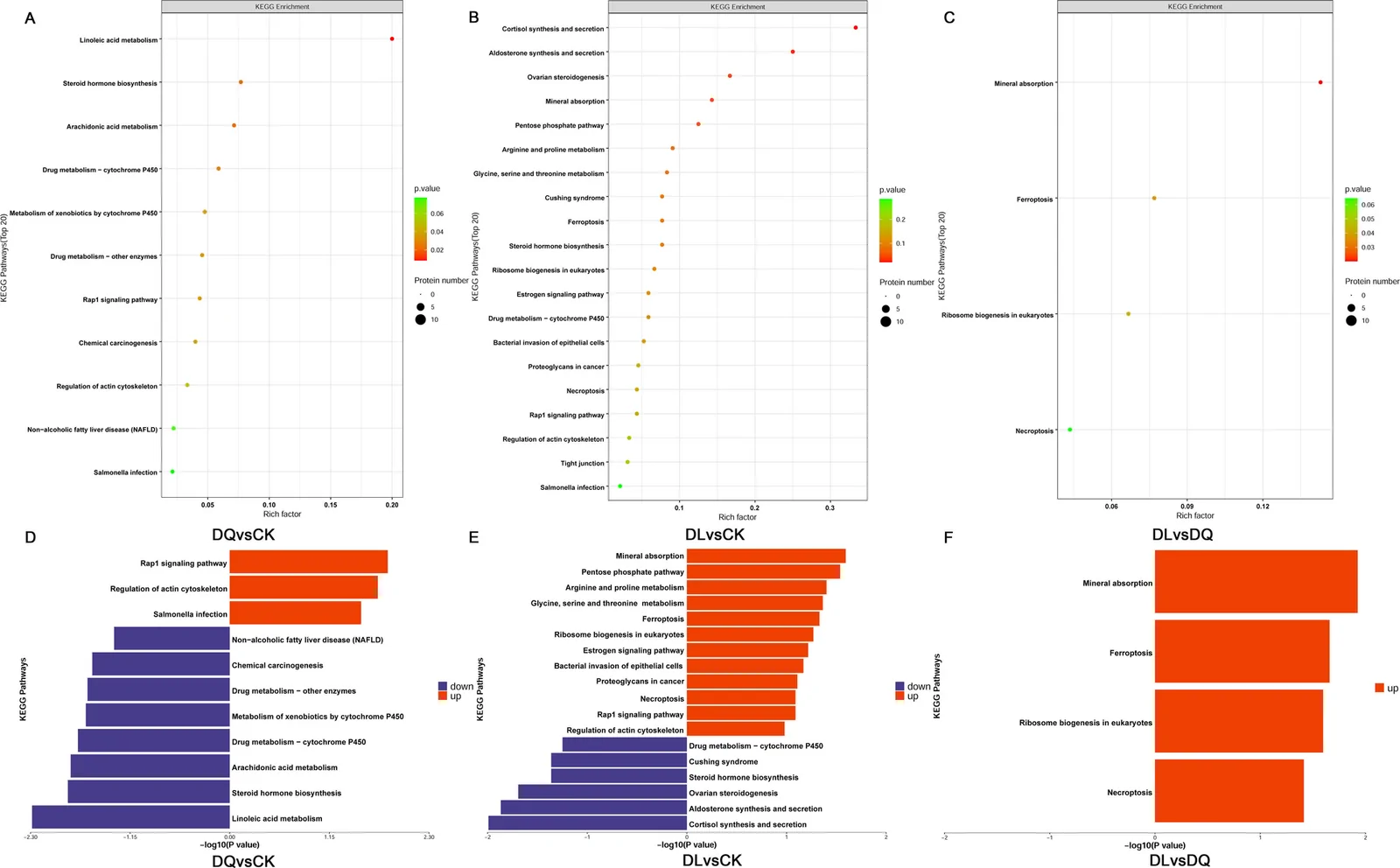
KEGG enrichment analysis of differentially expressed proteins (DEPs). **A-C** The size of the dots represents the number of DEPs, with the gradient from green to red indicating *p*-values, where lower values correspond to higher KEGG enrichment levels. **D-F** Enriched pathways of up- and down-regulated DEPs. CK, control group; DQ, diquat group; DL, diquat + LA group

**Fig. 4:**
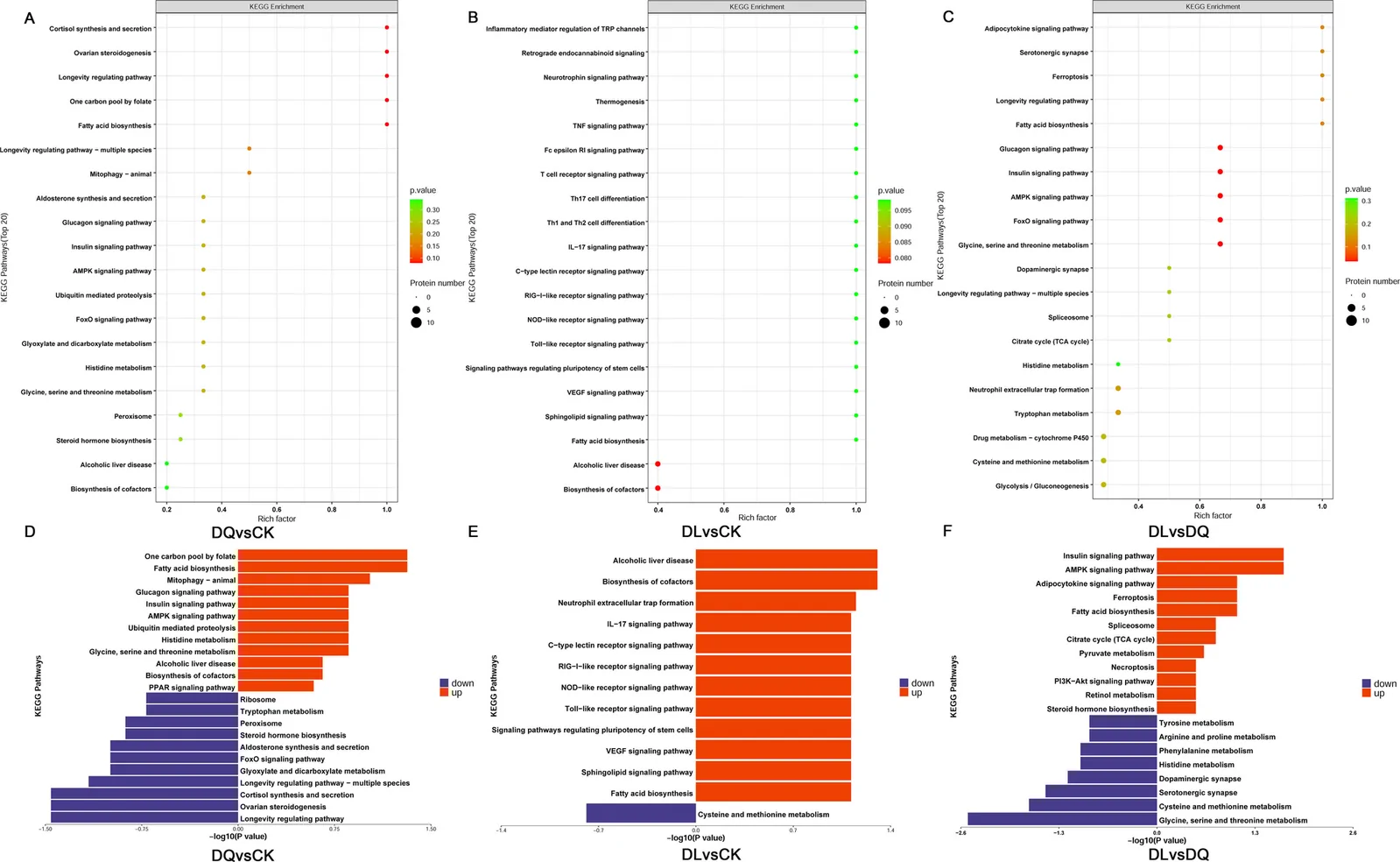
KEGG enrichment analysis of differentially ubiquitinated proteins (DUPs). **A-C** Dot size denotes the number of DUPs, with the gradient from green to red indicating *p*-values, where lower values correspond to higher KEGG enrichment levels. **D-F** Enriched pathways of up- and down-regulated DUPs. CK, control group; DQ, diquat group; DL, diquat + LA group

Additionally, the Kyoto Encyclopedia of Genes and Genomes (KEGG) annotations of DEPs and DUPs were used to explore the potential signaling pathways and metabolic processes by which LA regulates oxidative stress, and the enriched KEGG pathways terms are shown in Figs. 3A-F and 4A-F. For the comparison between the DQ and CK groups, the KEGG enrichment analysis showed that DEPs were significantly enriched in linoleic acid metabolism, steroid hormone biosynthesis, arachidonic acid metabolism, drug metabolism-cytochrome P450, etc. (Fig. 3A); up-regulated DEPs were mainly enriched in Rap1 signaling pathway, regulation of actin cytoskeleton, etc.; and down-regulated DEPs were mainly enriched in linoleic acid metabolism, steroid hormone biosynthesis, arachidonic acid metabolism, and drug metabolism-cytochrome P450 (Fig. 3D); DUPs were significantly enriched in lipid metabolism, amino acid metabolism, carbohydrate metabolism and signal transduction pathways (Fig. 4A); up-regulated DUPs were mainly enriched in glycine, serine and threonine metabolism, fatty acid biosynthesis, histidine metabolism, ubiquitin-mediated proteolysis, AMPK signaling pathway, etc.; while down-regulated DUPs were mainly enriched in longevity regulation pathway, ovarian steroidogenesis, cortisol synthesis and secretion, etc. (Fig. 4D). For the comparison between the DL and CK groups, KEGG enrichment analysis showed that DEPs were significantly enriched in cortisol synthesis and secretion, aldosterone synthesis and secretion, ovarian steroidogenesis and mineral absorption pathways (Fig. 3B); up-regulated DEPs were mainly enriched in glycine, serine and threonine metabolism, mineral absorption, pentose phosphate pathway, arginine and proline metabolism, ferroptosis, etc.; while down-regulated DEPs were enriched in cortisol synthesis and secretion, aldosterone synthesis and secretion, steroid hormone biosynthesis pathway, etc. (Fig. 3E); DUPs were significantly enriched in lipid metabolism, signal transduction, immune system, environmental adaptation, cell growth and death, amino acid metabolism, etc. (Fig. 4B); up-regulated DUPs were mainly enriched in biosynthesis of cofactors, alcoholic liver disease pathways, and other pathways; while down-regulated DUPs were mainly enriched in cysteine and methionine metabolism (Fig. 4E). The comparison between the DL and DQ groups via KEGG enrichment analysis revealed that DEPs were significantly enriched in pathways such as mineral absorption, ferroptosis, and ribosome biogenesis in eukaryotes (Fig. 3C); up-regulated DEPs were primarily enriched in mineral absorption, ferroptosis, and other pathways; while down-regulated DEPs showed on significant enrichment in relevant KEGG entries (Fig. 3F); for DUPs, significant enrichment was observed in glycine, serine and threonine metabolism, FoxO, AMPK, insulin, and glucagon signaling pathway, etc. (Fig. 4C); up-regulated DUPs were mainly enriched in FoxO, AMPK, and insulin signaling pathways, etc.; whereas down-regulated DUPs were primarily enriched in glycine, serine and threonine metabolism, tryptophan metabolism, and cysteine and methionine metabolism, etc. (Fig. 4F). These findings highlight that significant changes in ferroptosis, longevity regulation pathways, and AMPK signaling at both protein and ubiquitination levels are particularly noteworthy.

**Fig. 5:**
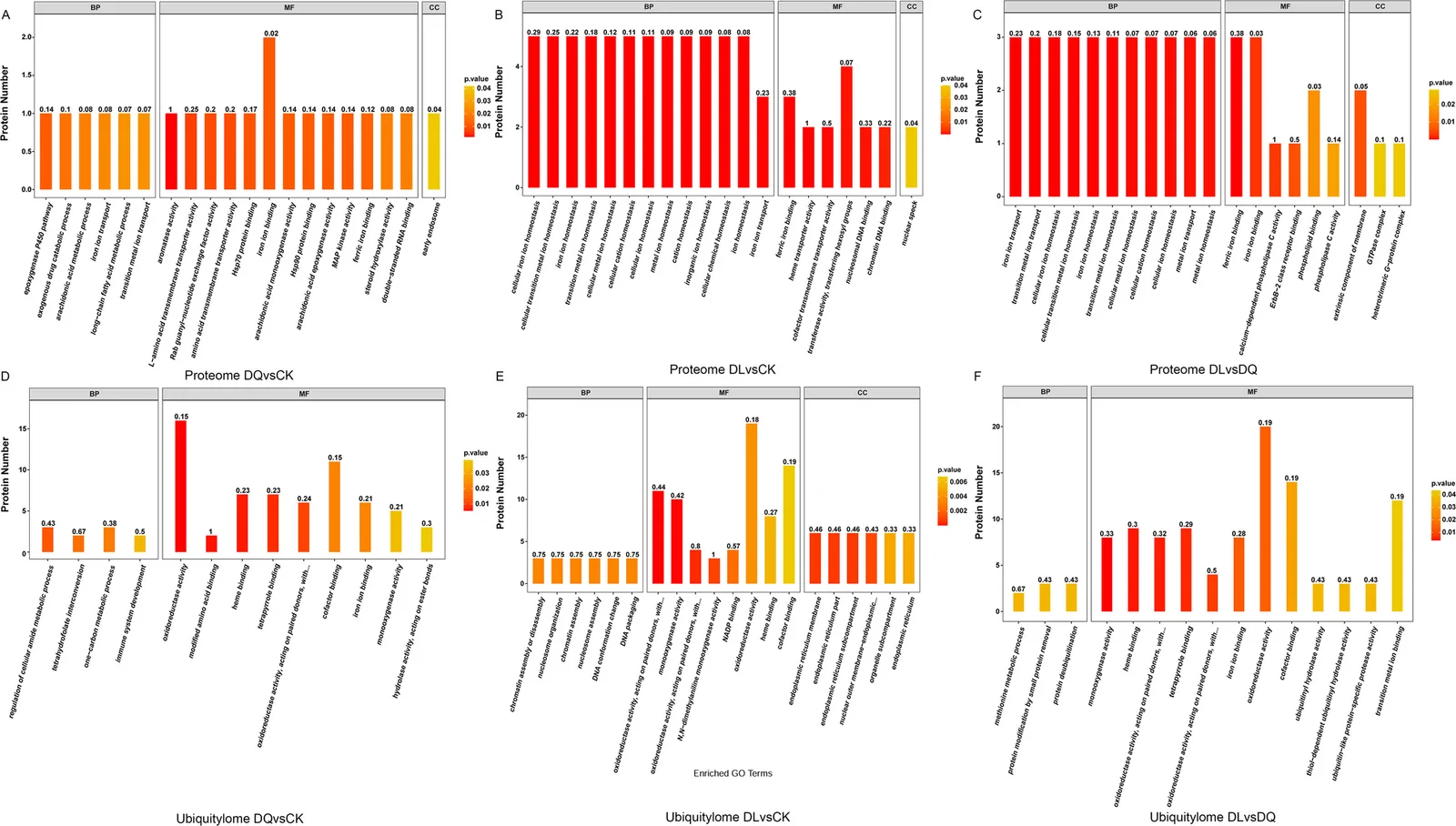
Top 20 enriched GO terms of differentially expressed proteins (DEPs, **A-C**) and differentially ubiquitinated proteins (DUPs, **D-F**). The x-axis shows the significantly enriched terms in the three GO categories: biological process (BP), molecular function (MF), and cellular component (CC). The y-axis represents the number of DEPs/DUPs enriched in these terms. Terms are sorted by *p*-value in ascending order, with the top 20 terms displayed. The number of terms shown for each category is determined by the proportion of significantly enriched terms in that category relative to the total. All terms are presented if fewer than 20 significantly enriched terms exist. Bar color indicates enrichment significance: a deeper red corresponds to a lower *p*-value and higher enrichment significance. The number above each bar is the rich factor (≤ 1), which represents the ratio of the number of DEPs annotated to the term to the total number of identified proteins annotated to the same term and is positively correlated with the degree of enrichment. CK, control group; DQ, diquat group; DL, diquat + LA group

Furthermore, to assess the regulatory effects of LA on physiological processes in the livers of oxidatively injured finishing pigs, especially during the recovery period, GO annotation and analysis were performed. All of the DEPs and DUPs were analyzed via GO enrichment in the biological process (BP), cellular component (CC) and molecular function (MF) categories. The GO enrichment analysis results are shown in Fig. 5A-F. For the comparison between the DQ group and CK groups, the main BP GO terms of DEPs were correlated with exogenous drug catabolic process, arachidonic acid metabolic process, iron ion transport, long-chain fatty acid metabolic process, etc.; the MF GO terms of DEPs were mainly related to amino acid transmembrane transporter activity, Hsp70 protein binding, iron ion binding, ferric iron binding, etc.; CC GO terms of DEPs were mainly enriched in early endosome (Fig. 5A); BP GO terms of DUPs mainly include immune system development, one-carbon metabolic process, and tetrahydrofolate interconversion; MF GO terms of DUPs were mainly enriched in iron ion binding, modified amino acid binding, oxidoreductase activity, etc. (Fig. 5D). For the comparison between the DL and CK groups, BP GO terms of DEPs were mainly related to iron homeostasis and iron ion transport; MF GO items of DEPs were mainly enriched in ferric iron binding, chromatin DNA binding and iron ion binding; CC GO terms of DEPs were mainly enriched in nuclear speck (Fig. 5B); the main BP GO terms of DUPs were DNA packaging, chromatin assembly or disassembly, etc.; MF GO terms of DUPs were mainly enriched in oxidoreductase activity and iron ion binding; CC GO terms of DUPs were mainly enriched in endoplasmic reticulum part, etc. (Fig. 5E). For the comparison between the DL and DQ groups, BP GO terms of DEPs were mainly correlated with iron ion transport, etc.; MF GO terms of DEPs were mainly enriched in iron ion binding, calcium-dependent phospholipase C activity, phospholipid binding, etc.; CC GO terms of DEPs were mainly associated with extrinsic component of membrane, heterotrimeric G-protein complex and GTPase complex (Fig. 5C); BP GO terms of DUPs were mainly related to methionine metabolic process, protein deubiquitination, and other terms; the main MF GO terms of DUPs were related to iron ion binding, oxidoreductase activity, ubiquitin-like protein specific protease activity, and ubiquitinyl hydrolase activity (Fig. 5F). Based on the above results, the enriched proteins identified in this study that are involved in iron ion binding and transport as well as oxidoreductase activity merit further exploration.

### Validation by parallel reaction monitoring (PRM)

**Fig. 6:**
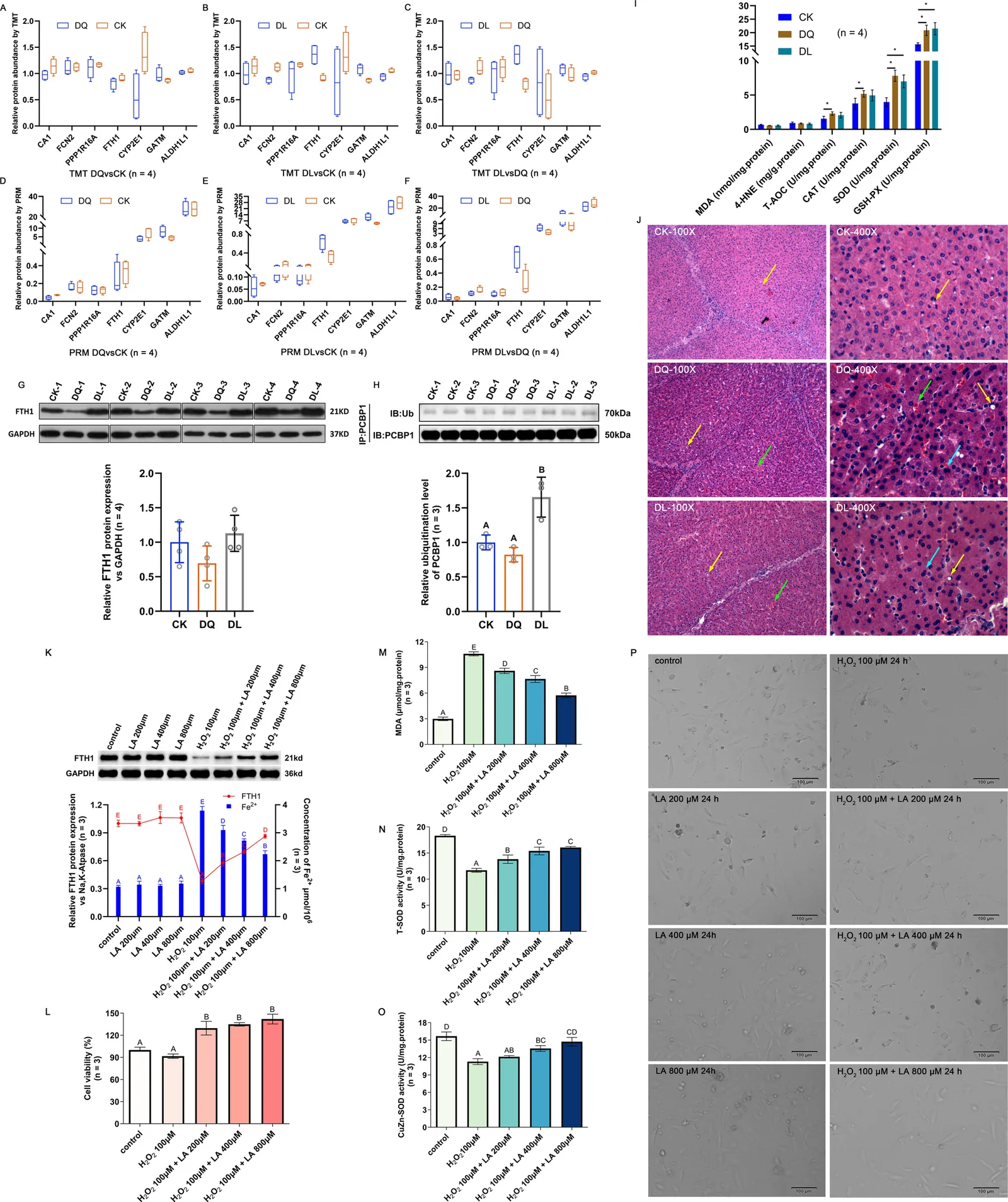
Expression patterns of candidate differentially expressed proteins (DEPs) quantified by tandem mass tag (TMT) and parallel reaction monitoring (PRM), and effects of LA on antioxidant capacity in oxidatively-stressed porcine liver and transformed human liver epithelial-2 (THLE-2) cells. **A-F** Relative abundance of selected proteins analyzed by TMT and verified by PRM, n = 4; **G** Western blot quantification of ferritin heavy chain 1 (FTH1) in porcine liver, n = 4; **H** Co-immunoprecipitation (Co-IP) analysis of poly(rC)-binding protein 1 (PCBP1) ubiquitination levels in porcine liver, n = 3; **I** Antioxidant status analysis of porcine liver, n = 4; **J** Hematoxylin–eosin (HE) staining of porcine liver sections; yellow arrows indicate microvacuoles; blue arrows indicate binucleated hepatocytes; green arrows indicate dilated and congested hepatic sinusoids; **K** Expression of FTH1 protein and concentration of Fe^2+^ in THLE-2 cells, n = 3; **L-O** Cell viability, malondialdehyde (MDA) content, total superoxide dismutase (T-SOD) and copper‑zinc superoxide dismutase (CuZn-SOD) activities in different groups, n = 3; **P** Micrographs of cell morphology. IB, immunoblotting; IP, immunoprecipitation; CAT, catalase; SOD, superoxide dismutase; GSH-Px, glutathione peroxidase; 4-HNE, 4-hydroxynonenal; T-AOC, total antioxidant capacity; values marked with * or different letters indicate significant differences among groups (*p* < 0.05)

As shown in Fig. 6A-F, the changes in protein abundance obtained by PRM showed similar trends between the DQ and CK groups (Fig. 6A and D), DL and CK groups (Fig. 6B and E), and DL and DQ groups (Fig. 6C and F) compared with the TMT analysis results, and the mass spectral information of the CYP2E1 protein is shown as an example (Additional file 3: Fig. S2 A-F). These results confirmed the reliability of the proteomics data.

### Quantitative Western blot validation of ferritin heavy chain 1 (FTH1) protein expression trends

As shown in Fig. 6G, consistent with the trend observed in proteomic screening, the hepatic FTH1 protein level was numerically higher in the LA supplementation group (DL group) than the oxidative injury group (DQ group). Although the statistical difference did not reach the conventional significance threshold (*p* = 0.054, marginal significance), the consistent upward trend matched the omics-based observation that dietary LA upregulated hepatic FTH1 expression during recovery after oxidative stress.

### Co‑immunoprecipitation (Co-IP) reveals that LA promotes poly(rC)-binding protein 1 (PCBP1) ubiquitination in porcine liver during recovery after oxidative stress

Consistent with ubiquitinome data, the Co-IP results demonstrated that the total ubiquitination level of immunoprecipitated PCBP1 was significantly increased in the DL group compared with the DQ group in porcine liver tissues collected during recovery after oxidative stress (Fig. 6H). This in vivo Co-IP assay directly verified the proteomic observation that dietary LA supplementation facilitated PCBP1 ubiquitination, strongly supporting the candidate ubiquitination targets screened from mass spectrometry data.

### Antioxidant status regulated by LA in porcine liver during recovery after oxidative stress

To investigate the effect of dietary LA supplementation on hepatic antioxidant capacity in oxidatively injured porcine liver, especially during the recovery period, we detected key antioxidant-related indicators (Fig. 6I). At this sampling stage, compared with the CK group, the DQ group showed a significant increase in total antioxidant capacity (T-AOC) levels (*p* < 0.05). The activities of catalase (CAT, *p* < 0.05), superoxide dismutase (SOD, *p* < 0.01), and glutathione peroxidase (GSH-Px, *p* < 0.01) were significantly elevated, indicating the hepatic antioxidant system may be excessively activated. Additionally, compared to the CK group, the DL group showed significantly increased SOD and GSH-Px activities (*p* < 0.01). Compared to the DQ group, the DL group showed no significant differences in most indicators (*p* > 0.05); except for GSH-Px, the levels of most indices in the DL group were intermediate between those in the CK and DQ groups, suggesting that LA supplementation contributed to the recovery of the overactivated hepatic antioxidant systems. Furthermore, hematoxylin and eosin (HE) staining of liver sections (Fig. 6J) showed that during the oxidative stress recovery period, all groups exhibited regular hepatic plate arrangement, with no obvious hepatocyte necrosis or inflammatory cell infiltration. The livers of oxidative stress-challenged pigs showed increased microvacuoles, several binucleated hepatocytes, and mild sinusoidal dilation and congestion, indicating a certain degree of damage at this sampling stage, which was alleviated by LA supplementation.

### Regulation of antioxidant levels by LA in transformed human liver epithelial-2 (THLE-2) cells under oxidative stress

The results showed that LA at concentrations of 200, 400, and 800 μM significantly alleviated (*p* < 0.01) the H₂O₂-induced significant decrease in FTH1 protein expression level (*p* < 0.01) and significant increase in Fe^2+^ concentration (*p* < 0.01) (Fig. 6K). Different concentrations of LA significantly alleviated (*p* < 0.01) the H₂O₂-induced significant reduction in THLE-2 cell viability (*p* < 0.01, Fig. 6L) and significant increase in malondialdehyde (MDA, *p* < 0.01, Fig. 6M). LA at 400 and 800 μM significantly relieved H₂O₂-induced significant decrease in T-SOD and CuZn-SOD activities (*p* < 0.01, Fig. 6N-O). Microscopic observation showed that the number of THLE-2 cells treated with 100 μM H₂O₂ was significantly reduced, while LA treatment effectively inhibit H₂O₂-induced cell death (Fig. 6P).

Notably, primary porcine hepatocytes suffer from cumbersome isolation, low yield, restricted passaging and poor stability, so we adopted THLE-2 human hepatocytes for in vitro tests. This cell line retains complete hepatic antioxidant and iron homeostasis pathways with stable culture performance and is widely used for hepatotoxicity evaluation in existing studies. Nevertheless, cross-species differences exist between human THLE-2 cells and our porcine in vivo model. Subsequent work will adopt primary porcine hepatocytes for homologous verification.

## Discussion

### DQ-induced hepatic oxidative injury and the regulatory effect of LA

According to the analysis results of growth performance and plasma antioxidant level data of pigs in this study (Additional file 3: Fig. S3), compared with the control group (CK group), intraperitoneal instillation of diquat (DQ group) significantly decreased both the average daily feed intake (ADFI) and average daily weight gain (ADG) of pigs (*p* < 0.01), and SOD, CAT and GSH-Px activities in plasma decreased significantly (*p* < 0.01), MDA level increased significantly (*p* < 0.01); and dietary LA supplementation alleviated these trends. Thus, it can be seen that diquat can significantly impaired the production performance and systemic antioxidant capacity in pigs, while dietary LA improved the growth performance and antioxidant status under oxidative stress (Bao et al. 2016).

Diquat is a bipyridine herbicide that uses molecular oxygen to produce superoxide anion radical followed by hydrogen peroxide (H_2_O_2_) (Sewalk et al. 2001), the production of which is considered a key mechanism for diquat induced cytotoxicity (Zhou et al. 2024; Qiu et al. 2025). Normally, cells protect themselves from ROS damage through intracellular antioxidant enzymes, including T-SOD, GSH-Px, CAT, etc. Numerous studies have shown that diquat can reduce the activity of antioxidant enzyme in liver (Sewalk et al. 2001; Han et al. 2025). Nevertheless, we analyzed liver samples obtained on the 15th day after a single diquat treatment and found elevated antioxidant levels at this sampling stage, which appears inconsistent with the findings of some prior studies. In fact, these results are of great reference significance and deserve further consideration and exploration. Earlier studies have also reported that paraquat, another bipyridine compound, can significantly increase SOD activity in the liver of carp (Víg and Nemcsók 1989). Other investigations found that paraquat can increase GSH-Px activity in erythrocytes of carp (Gabryelak and Klekot 1985). The underlying reasons are analyzed as follows.

On the one hand, this phenomenon is not contradictory to previous studies, which may be attributed to the temporal differences induced by varying sampling time points. Some studies have shown that the changes in antioxidant enzyme levels in animals may fluctuate after oxidative stress; that is, the changes in antioxidant enzyme levels may occur alternately at different stages (Ying Wang et al. 2018). In a study investigating the effects of chronic diquat exposure on antioxidant levels in zebrafish liver, the SOD level increased significantly in the first 14 days (*p* < 0.05), and a downward trend emerged on the 28th day (*p* > 0.05), while MDA content increased significantly on the 28th day after diquat treatment (Shen et al. 2021). The concentration of diquat in serum and tissues decreased rapidly after acute diquat poisoning, and the duration of the recovery phase differs across studies. Some studies found that the body weight of rats initially declined in rats suffering from acute diquat poisoning and recovered to the level before poisoning on the 14th day, whereas the tissue damage of liver was still not fully resolved (Tuokang et al. 2020), which was basically consistent with the pattern observed in this study.

On the other hand, this result does not indicate that diquat failed to induce oxidative stress, but rather reflects that after acute oxidative shock, the body enters a late phase of recovery following the discontinuation of harmful stimulation and involving a compensatory upregulation mechanism of the antioxidant system (Lei et al. 2016; Acar 2021). At this stage, the body compensatorily activates the antioxidant signaling pathways to significantly upregulate the expression of antioxidant enzymes and even produce abundant antioxidants in response to toxic stimulation; these changes efficiently scavenge lipid peroxides and rapidly eliminate toxic metabolites. A series of studies have demonstrated that under oxidative stress, excessive reactive oxygen species (ROS) can trigger Nrf2-mediated responses. Under normal conditions, Nrf2 binds to its inhibitor Kelch-like ECH-associated protein 1 (Keap1) and remains inactive in the cytoplasm. Upon stimulation by stressors such as ROS, Nrf2 dissociates from Keap1 and upregulates the transcription of antioxidant genes (Zheng et al. 2016; Zhu et al. 2018). Accordingly, when SOD activity is increased, the elevated H₂O₂ levels accompanied by oxidative stress trigger the upregulation of CAT and/or GSH-Px, which ultimately acting as a compensatory defense strategy through the maintenance of antioxidant balance (Rosa et al. 2021). Moreover, high concentrations of MDA and 4-hydroxynonenal (4-HNE) can rapidly undergo cross-linking reactions with proteins and DNA to form stable adducts (Winczura et al. 2012), making these bound adducts difficult to detect. All the above factors further contribute to the phased enhancement of in vivo antioxidant capacity, ultimately resulting in a decreasing trend in MDA and 4-HNE content and a compensatory enhancement of antioxidant system function.

Furthermore, most of the indicators in the DL group were numerically between those in the DQ and CK groups. These results suggest that dietary supplementation with LA can alleviate the excessive oxidative stress and severe compensatory activation induced by diquat to a certain extent, promoting a mild regression of hepatic antioxidant status towards the normal physiological level without generating a significant reversal effect. This mild regulatory pattern is closely related to the long sampling cycle of this study and fully reflects the dynamic temporal pattern of the occurrence, development, and repair of oxidative stress. Meanwhile, it confirms that lipoic acid can exert hepatic protective effect by buffering the excessive stress response and maintaining the organism's antioxidant homeostasis. Admittedly, this phenomenon still requires confirmation and mechanistic interpretation in further research.

### LA affects liver antioxidant levels by regulating the expression and ubiquitination of key proteins in ferroptosis pathway

**Fig. 7:**
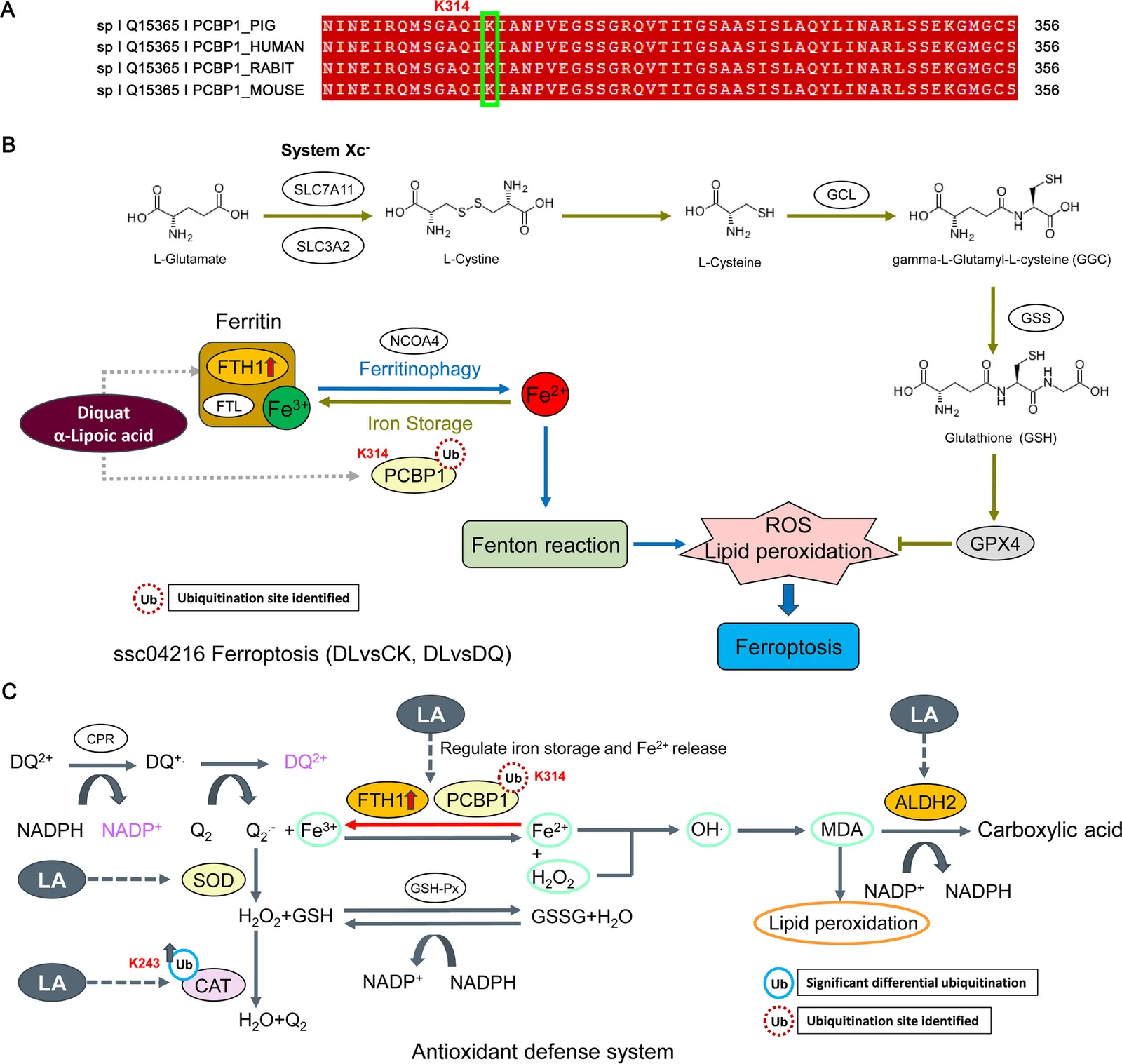
Potential mechanism of α-lipoic acid (LA) in regulating porcine hepatic antioxidant capacity through the ubiquitination of key ferroptosis-related proteins. **A** Alignment of amino acid sequences of poly(rC)-binding protein 1 (PCBP1) across species; **B** LA‑mediated regulation of the expression and ubiquitination of core proteins in the ferroptosis pathway in porcine liver; **C** Mechanism of diquat-induced oxidative stress and the LA‑mediated regulation of the expression and ubiquitination of related enzymes. DQ, diquat; FTH1, ferritin heavy chain 1; FTL, ferritin light chain; NCOA4, nuclear receptor coactivator 4; GPX4, glutathione peroxidase 4; GSS, glutathione synthase; GCL, glutamate cysteine ligase; system Xc^−^, cystine/glutamate antiporter system; SLC7A11, solute carrier family 7 member 11; SLC3A2, solute carrier family 3 member 2; ROS, reactive oxygen species; SOD, superoxide dismutase; CAT, catalase; GSH-Px, glutathione peroxidase; GSH/GSSG, glutathione (Reduced/Oxidized); NADP^+^/NADPH, nicotinamide adenine dinucleotide phosphate (Oxidized/Reduced); MDA, malondialdehyde; ALDH2, aldehyde dehydrogenase 2; CPR, cytochrome P450 reductase; ${\mathrm{O}}_{2}^{\cdot -},$ superoxide anion radical; ssc04216, KEGG pathway ID. Upward arrows (↑) denote increased protein abundance or elevated ubiquitination levels. K243 and K314 are ubiquitination sites of CAT and PCBP1, respectively. Ub in red dashed circles denotes proteins exhibiting de novo ubiquitination upon LA treatment; Ub in blue solid circles denotes proteins with altered ubiquitination levels under LA intervention

This study identified FTH1 (a DEP enriched in the ferroptosis pathway) and PCBP1 (a DUP with ubiquitination at lysine site K314). FTH1 was upregulated and PCBP1 displayed modified ubiquitination in oxidatively injured porcine liver during the recovery period with dietary LA supplementation (DL group, Fig. 7A-B). Amino acid sequence alignment revealed that the ubiquitinated lysine site K314 on PCBP1 screened in this experiment is highly conserved across species, suggesting that this protein and its K314 residue exert important and conserved biological functions (Fig. 7A). According to proteomic analysis results, hepatic FTH1 protein expression was significantly upregulated in oxidatively injured porcine liver during the recovery period from pigs receiving dietary LA supplementation (DL group) compared with the CK and DQ groups. This trend was further verified via PRM quantification and Western blot analysis, forming a complete evidence chain together with the proteome data. Furthermore, cell-based assays further verified that LA significantly ameliorated the reduction in FTH1 and the elevation in Fe^2+^ concentration induced by oxidative stress, and the protective effect of LA exhibited a concentration‑dependent manner within a certain range.

Iron is an important element, but excess iron generates cytotoxic ROS through Fenton reaction and induces ferroptosis; thus, it is important to maintain cellular iron homeostasis (Jiang et al. 2021; Li et al. 2024a, b). The liver is the main site of iron storage in mammals, and it is also the primary organ regulating iron balance through the synthesis and secretion of iron regulatory hormone-hepcidin (Li et al. 2024a, b; Fisher et al. 2025; He et al. 2025). Combined with the mechanism by which diquat induces oxidative stress (Fig. 7C), the liberation of iron ions in ferritin plays an important role in diquat-induced cytotoxicity, while enzymes such as CAT and GSH-Px play exert protective functions in this process. In this study, ubiquitination of CAT and other enzymes was also observed in porcine liver exposed to diquat-induced oxidative stress under LA treatment. We speculate that LA may modulates diquat-induced oxidative stress by regulating the ubiquitination of key proteins and oxidoreductases of ferroptosis pathway, affecting the storage and release of iron ions as well as the production of H_2_O_2_. The effect of ubiquitination on the expression and activity of related enzymes requires further investigation.

Hua et al. found that diquat can obviously induce ferroptosis in hepatocytes (Hua et al. 2022). Ferroptosis is characterized by the accumulation of lipid peroxidation products and ROS from iron metabolism, and is associated with multiple physiological and pathological processes such as cancer, neurotoxicity, cellular immunity, etc. (Xie et al. 2016). In the ferroptosis pathway (Fig. 7B), FTH1 constitutes functional ferritin together with ferritin light chain (FTL). As the core functional subunit of ferritin, FTH1 converts Fe^2+^ into Fe^3+^ and sequesters iron within ferritin through its ferritin peroxidase activity to inhibit the Fenton reaction, thereby maintains cellular iron homeostasis, suppresses Fenton reaction-mediated ROS production, and renders FTH1 a key defensive protein against ferroptosis (Arosio et al. 2009; Chen et al. 2022a, b; Galy et al. 2024). When the intracellular iron level is low, ferritin is degraded through the autophagy pathway of nuclear receptor coactivator 4 (NCOA4), leading to the release of iron from lysosomes into the cytoplasm (Mancias et al. 2014). On the other hand, intracellular glutamate can be exchanged for extracellular cysteine by the Cystine/glutamate antiporter system (system Xc^−^) consisting of SLC7A11 and solute carrier family 3 member 2 (SLC3A2), thus synthesizing glutathione (GSH); while GPX4 utilizes the antioxidant property of GSH to detoxify lipid hydrogen peroxide, thus inhibiting ferroptosis and protecting cells (Ohta et al. 2021). Theoretically, a decrease in FTH1 would lead to excess unstable iron production, which in turn would promote the formation of oxidative free radicals (Scaramuzzino et al. 2021). It has been shown that overexpression of FTH1 significantly inhibits cisplatin-mediated increase in intracellular ROS, while knockout of FTH1 enhances cisplatin-mediated increase in ROS (Salatino et al. 2019). In addition, FTH1 knockout promotes apoptosis induced by human parainfluenza virus 2 (hPIV-2) (Ohta et al. 2021). In this study, LA increased FTH1 expression in the liver of oxidatively stressed pigs, and this trend was further verified by PRM protein quantitative analysis (Fig. 6A-F) and cell-based assays. Therefore, we infer that ferroptosis serves as a key pathway through which LA modulates hepatic antioxidant capacity in oxidatively injured porcine liver, especially during the recovery period.

PCBP1 is an iron chaperone that can coordinate iron via its cysteine residues and regulate iron metabolism through glutamate residues and non-covalently bound GSH, thereby binding iron and delivering it to ferritin, a cytosolic iron storage protein, and playing important roles in the storage, utilization, and export of Fe^2+^ (Nandal et al. 2011; Ryu et al. 2017; Patel et al. 2019; Galy et al. 2024; Artusi et al. 2025). The present study demonstrated that mice with hepatic PCBP1 deletion exhibited iron imbalance, characterized by decreased hepatic iron stores and elevated labile iron levels. This imbalance triggered ROS generation, induced lipid peroxidation and ferroptosis, and ultimately promoted the development of chronic liver disease in mice (Jadhav et al. 2021). Another study showed that the natural product acevaltrate (ACE) directly binds to the iron chaperones PCBP1/2 and downregulates their expression, leading to a rapid increase in intracellular Fe^2+^ levels, and it also targets GPX4 and promotes its degradation via the ubiquitin–proteasome pathway, thereby reducing the accumulation of lipid peroxidation (Yu et al. 2025). In this study, LA modulated the ubiquitination of PCBP1 in oxidatively injured porcine liver, especially during the recovery period. Meanwhile, Co-IP assays confirmed that LA increased the ubiquitination level of hepatic PCBP1 in the DL group. However, the conclusions drawn in the present study have certain limitations. Systematic functional verification of the K314 ubiquitination site in iron metabolism and ferroptosis phenotypes will constitute the core focus of our subsequent independent research. Moreover, the specific effects of this ubiquitination on the activity and biological function of PCBP1 remain to be further explored (Protchenko et al. 2020).

### Inferred protein interaction network of ferroptosis pathway from omics enrichment data

KEGG enrichment identified the ferroptosis pathway as a markedly enriched signature in the DL vs. DQ comparison, while GO terms centered on iron homeostasis, iron ion transport and ferric iron binding were repeatedly enriched at both protein expression and ubiquitination levels. These enriched functional modules reflect a coordinated regulatory network formed by iron transport proteins, iron-binding oxidoreductases and ferroptosis executors.

Functionally, iron transport proteins, iron-binding oxidoreductases and ferroptosis-related functional proteins interact synergistically to limit abnormal intracellular iron accumulation: iron ion transporters (e.g., solute carrier family members involved in mineral absorption) mediate the efflux of excess cytoplasmic iron, while iron-binding oxidoreductases including ferric reductases, SOD family proteins and cytochrome P450 enzymes maintain cellular redox homeostasis via ferric/ferrous iron binding, and these two types of protein modules jointly suppress ferroptosis signal transduction. Under diquat-induced oxidative stress (DQ group), the ubiquitination levels of iron ion-binding proteins were suppressed, accompanied by impaired iron ion transport capacity according to GO enrichment data. Disrupted iron trafficking breaks intracellular iron homeostasis, promotes excessive ferric iron accumulation and triggers ferroptotic liver injury, which aligns with the mild hepatic pathological damage observed in HE-stained liver sections collected at the recovery stage.

Notably, the present multi-omics data implied that dietary LA remodels the ubiquitination status of ferroptosis-related proteins (especially PCBP1) to alleviate oxidative stress and ferroptosis in diquat-challenged porcine liver, which constitutes the core innovation of the current study. Moreover, the AMPK and FoxO signaling pathways, which were prominently enriched in the ubiquitinome dataset, may function as upstream regulatory hubs to orchestrate this iron-centered homeostasis network. Mechanistically, activated AMPK further triggers downstream transcriptional programming via FoxO, which governs the expression of E3 ubiquitin ligases (e.g., Parkin and HUWE1) (Lu et al. 2025), and subsequently modulates the abundance of iron homeostasis executors including PCBP1 and FTH1 (Wu et al. 2022), thereby coordinating iron trafficking, iron storage and ferroptosis signaling at the transcriptional and post-translational levels. Based on the ubiquitinome enrichment signatures, we prospectively speculate that unidentified upstream E3 ubiquitin ligases and deubiquitinases (DUBs) act as critical mediators governing LA-induced ubiquitination of PCBP1. These undiscovered ligases and deubiquitinases are predicted to dynamically control the ubiquitination level of PCBP1, further tuning its iron-binding function and downstream ferroptosis signal transduction. This prospective regulatory axis (E3 ligase/DUB–PCBP1/FTH1–ferroptosis) deduced from omics data will be the core content of our subsequent independent research.

## Conclusions

In this study, we integrated proteomic and ubiquitylomic approaches to explore the mechanism by which LA regulates hepatic antioxidant function via modulating the expression and ubiquitination of key proteins involved in the ferroptosis pathway. Preliminary validations were performed using PRM quantification, histopathological analysis, and cell-based assays. DEPs and DUPs identified in the liver of LA-supplemented oxidatively stressed pigs were closely associated with the ferroptosis pathway. In porcine liver tissues during recovery after oxidative stress, LA upregulated FTH1 abundance and promoted the ubiquitination of PCBP1. Consistent with in vivo findings, LA significantly ameliorated the reduction in FTH1 protein expression and the elevation in Fe^2+^ concentration induced by oxidative stress in THLE-2 cells. FTH1 and PCBP1 play critical roles in the storage of iron in the ferroptosis pathway. The sequestration of excess intracellular iron attenuates lipid peroxidation and subsequent ferroptosis driven by the Fenton reaction, thereby protecting hepatocytes. In addition, a key ubiquitination residue (K314) was identified on PCBP1. By identifying FTH1 and PCBP1 as core molecular targets of LA, this study establishes a functional link between the ubiquitination and ferroptosis pathways in hepatic antioxidant regulation. Furthermore, dietary LA effectively balanced the compensatory activation of the antioxidant system and alleviated oxidative stress-induced liver injury during the oxidative stress recovery period. Further studies are required to verify the specific functions of FTH1 and PCBP1 expression and ubiquitination in LA-mediated hepatic antioxidant regulation, clarify the functional influence of K314 ubiquitination on PCBP1, and identify the upstream E3 ubiquitin ligases and deubiquitinases responsible for LA-induced PCBP1 ubiquitination. Collectively, this study provides a theoretical basis for the application of LA as a feed additive to improve livestock liver health from the perspective of protein post-translational modification.

## Methods and materials

### Experimental animals and sample collection

18 castrated male Large White (LW) pigs with an initial body weight of 70.64 ± 3.61 kg were randomly divided into 3 groups (n = 6) to avoid subjective bias during grouping. Each pig was housed individually in 160 cm ×90 cm ×120 cm metabolic cages, provided with ad libitum access to water, and fed a fattening diet (Additional file 4: Table S1). Each group of pigs were fed with formulated diet containing 0 mg LA/kg diet (CK, control group), 0 mg LA/kg diet plus diquat challenge (DQ, diquat group), and 800 mg LA/kg diet plus diquat challenge (DL, diquat + α-lipoic acid group), respectively. On the 15th day of the experiment, DQ and DL groups were treated with a one-time dose of diquat (8 mg/kg body weight) via intraperitoneal injection, and the CK group received an equal amount of physiological saline via one-time intraperitoneal injection. Liver samples were collected on the 29th day to analyze the regulatory effect of dietary addition of LA supplementation on the expression and ubiquitination of key proteins in oxidatively-stressed porcine liver. During the trial, pigs were carefully managed by dedicated staff to minimize extraneous stressors unrelated to the experiment. The pigs were fed three times daily at 07:00, 12:00 and 18:00, with feeding amounts adjusted to leave a small quantity of residual feed after each meal. The ambient temperature was maintained at 15–20 °C, and relative humidity was kept between 40% and 60%. Diquat dibromide monohydrate (DQ, 45,422-250MG-R, Sigma-Aldrich) was dissolved in 250 mL normal saline for intraperitoneal injection. DL-α-lipoic acid (LA, Cat. No. Y0000546, Sigma-Aldrich) was supplemented to the basal diet at a dosage of 800 mg/kg. Blinding was implemented throughout the experiment. All liver sample processing and subsequent omics and Western blot analyses were conducted by researchers unaware of treatment grouping.

Immortalized human liver epithelial THLE-2 cells (ATCC CRL-2706, RRID:CVCL_3803) were purchased from Meisen CTCC (Zhejiang Meisen Cell Technology Co., Ltd., China; internal catalog No. CTCC-004–0030, Lot No. M21SJ1304). Cells were cultured in THLE-2 complete medium (Meisen CTCC, Cat# CTCC-002–046) in a humidified incubator at 37 °C with 5% CO₂, with culture flask caps loosely tightened to allow gas exchange. The medium was changed every 1–2 days. When the cells reached 80% confluence, they were digested and passaged. After trypsin digestion, the cell density was adjusted to 5 × 10^4^ cells/mL and seeded into 96-well plates at 100 μL per well. LA (purity 99.56%, CAS No. 1200–22-2, MCE, USA) was dissolved in DMSO to prepare solutions with concentrations of 0, 200, 400, and 800 μM, followed by cell treatment for 24 h. The cells were then further incubated with 100 μM H₂O₂ for an additional 24 h. The STR identification certificate, COA, and mycoplasma testing reports of THLE-2 cells are provided in Additional file 5. The detailed screening procedures for LA concentrations, H₂O₂ concentrations and treatment durations are provided in Additional file 6.

### Measurement of antioxidation indices in the liver of finishing pigs

The levels of 4-HNE and MDA were detected by ELISA and colorimetry, respectively, using commercial assay kits according to the manufacturer’s instructions (DKM-L0027, DKM-60003, Beijing Dakemei Technology, China). The activities of SOD, CAT, T-AOC, and GSH-Px were detected via colorimetry using corresponding commercial kits according to the manufacturer’s instructions (DKM-60001, DKM-60015, DKM-60021, DKM-60005).

### TMT-based quantitative proteomics analysis and PRM validation

Preliminary quality control was conducted to evaluate total hepatic protein extraction efficiency, consistency of protein concentration and sample protein integrity (Additional file 3, Fig. S4). On this basis, four out of six biological replicates per group were randomly selected for proteomic and ubiquitylomic analyses. For TMT-based quantitative proteomics analysis, hepatic tissues were extracted using the SDT cleavage method (4% [w/v] SDS, 100 mM Tris–HCl, 1 mM dithiothreitol [DTT], pH 7.6) and quantified with the BCA Protein Assay Kit (Bio-Rad, USA); 200 μg of protein per sample was trypsinized via the filter-aided sample preparation (FASP) method. 40 μL of 0.1% formic acid was added to the freeze-dried peptides for peptide quantification (OD280), and 100 μg of the peptide mixture was labeled with TMT reagent (Thermo Scientific, USA) according to the manufacturer’s instructions. Labeled peptides from each group were mixed in equal amounts and fractionated using the High pH Reversed-Phase Peptide Fractionation Kit (Thermo Scientific), involving column equilibration with acetonitrile and 0.1% trifluoroacetic acid (TFA), sample loading, low-speed centrifugation for desalting, gradient elution of column-bound peptides with high-pH acetonitrile solution, and vacuum drying of each peptide sample. LC–MS/MS analysis was performed on a Q Exactive HF mass spectrometer (Thermo Scientific) coupled to Easy nLC (Proxeon Biosystems, now Thermo Fisher Scientific) for 60 min with a gradient elution program (0 − 3 min, 5 − 8% solvent B [84% acetonitrile and 0.1% formic acid]; 3 − 43 min, 8 − 26% solvent B; 43 − 48 min, 26 − 40% solvent B; 48 − 50 min, 40 − 90% solvent B; 50 − 60 min, 90% solvent B); MS raw data were searched using the MASCOT engine (version 2.2, Matrix Science, UK) embedded in Proteome Discoverer 1.4 software for protein identification and quantitation (Additional file 4: Table S2), followed by calculation of protein abundance ratios and Student’s *t*-test, and PRM validation was performed on a Q Exactive HF mass spectrometer (Thermo Scientific).

### Database search and bioinformatics analysis

The protein sequences of selected DEPs were aligned using NCBI BLAST + tools, and GO functional annotation was performed via Blast2GO software (http://www.blast2go.com/). KEGG pathway enrichment was conducted based on the official KEGG database (https://www.kegg.jp/). Enrichment analyses were performed via Fisher’s exact test, with all quantified proteins as the background dataset; *p-values* were adjusted using the Benjamini–Hochberg correction for multiple testing, and only functional categories and pathways with adjusted* p* < 0.05 were deemed significant. Protein subcellular localization was predicted with CELLO (http://cello.life.nctu.edu.tw/). InterProScan software was used to scan the InterPro database for sequence functional characterization, thus acquiring Pfam database-derived domain annotation information of the target protein sequences.

### Ubiquitylome analysis based on label-free technique

Label-free quantitative ubiquitylomics analysis was performed as follows. Liver samples were lysed in UA buffer (8 M urea, 100 mM Tris/HCl, pH 8.5), and protein concentrations were determined using a Bradford protein assay kit (Sigma-Aldrich). Extractable proteins were verified by SDS-PAGE. The proteins were reduced with 10 mM DTT at 37 °C for 1.5 h with shaking at 600 rpm, alkylated with 50 mM iodoacetamide (IAA) in the dark for 30 min, and then diluted with 4 volumes of 50 mM Tris–HCl (pH 8.0) to reduce the urea concentration to 2 M. Tryptic digestion was performed at a protein:trypsin mass ratio of 50:1 at 37 °C overnight (15–18 h). TFA was added to a final concentration of 0.1% to adjust the pH to ≤ 3. After lyophilization, peptides were reconstituted in pre-cooled immunoaffinity purification (IAP) buffer and enriched using pretreated anti-K-ε-GG antibody beads (PTMScan ubiquitin remnant motif K-ε-GG kit, Cell Signaling Technology) at 4 °C for 1.5 h. The beads were washed three times with IAP buffer and three times with pre-cooled water, then eluted with 0.15% TFA. LC–MS/MS analysis was carried out on a timsTOF Pro mass spectrometer (Bruker, Germany) coupled to a Nanoelute system (Bruker Daltonics) at a flow rate of 300 nL/min using a gradient elution program. MS raw data were processed and searched using MaxQuant software (version 1.6.14.0) for protein identification and quantification (Additional file 4: Table S3).

Raw proteomic and ubiquitylomic data were deposited in iProX, an official member repository of the ProteomeXchange Consortium (Ma et al. 2019, Chen et al. 2022c).

### FTH1 expression in the liver detected by Western blot

Liver samples were homogenized in RIPA lysis buffer (WB0003, Dalmei, China) containing protease inhibitors and 1 mM PMSF (0754, Amresco, USA) at a tissue weight-to-buffer ratio of 1:9. After centrifugation at 13,000 rpm (20 min), supernatants were diluted to 4 mg/mL with 5 × loading buffer and denatured at 100 °C. Equal amounts of protein (20 μg per lane) were separated by SDS-PAGE using a 5% stacking gel and 12% separating gel, then transferred to 0.45 μm NC membranes (HATF00010, Millipore, USA). Membranes were blocked with 3% BSA in TBST, incubated overnight with anti-FTH1 primary antibody (ab231253, Abcam, UK, 1:10,000), and incubated with anti-GAPDH primary antibody (1:10,000) for 2 h as the internal reference. After TBST washing, membranes were incubated with HRP-conjugated secondary antibody (S004, Dalmei, China, 1:10,000) for 40 min. Protein bands were visualized with ECL reagent (WBKLS0500, Millipore, USA) on X-ray films. Band integrated optical density was quantified using ImageJ and Total Lab Quant V11.5 software.

### Co-IP detection of PCBP1 ubiquitination in liver tissue

Liver total protein was extracted using RIPA lysis buffer containing protease and deubiquitinase inhibitors. Equal amounts of total protein (800 μg) were incubated with anti-PCBP1 antibody (YA2927, MedChemExpress, USA) at 4 °C overnight, followed by incubation with protein A/G magnetic beads (HY-K0202, MedChemExpress, USA) to enrich immunoprecipitated PCBP1. After repeated washing, bound proteins were eluted by 1 × Loading buffer and separated by SDS-PAGE. The ubiquitination level of PCBP1 was sequentially detected using PCBP1 antibody and P4D1 antibody (3936 T, Cell Signaling Technology, USA). Band gray values were measured via ImageJ, and the relative ubiquitination level was calculated as P4D1 gray intensity to PCBP1 gray intensity.

### Preparation of liver tissue slices and HE staining

According to the technical requirements of HE staining, porcine liver tissue was fixed in 4% paraformaldehyde for 72 h, and after gradient dehydration with ethanol, the tissue was processed for paraffin embedding, and the tissue wax block were cut into 4‑μm‑thick sections with a microtome. Paraffin sections were dewaxed with xylene for 5 min, rehydrated with gradient ethanol, then stained with hematoxylin for 4 min, differentiated by 1% hydrochloric alcohol, immersed in eosin for 3 min, dehydrated with gradient ethanol, cleared with xylene, and finally mounted with neutral gum. Images were collected under a microscope for analysis of liver morphology.

### Detection of antioxidant status and iron metabolism in THLE-2 cells

Cells from each group were collected and detected according to the instructions of the CCK-8 assay kit (CK04, DoJinDo, Japan). Briefly, 10 μL of CCK-8 solution was added to each well. After 4 h of incubation, the absorbance of each well at 450 nm was measured by a microplate reader to calculate cell viability. Cell morphology was observed under a microscope. According to the manufacturer’s protocols (E-BC-K025-M, E-BC-K022-Mt, E-BC-K881-M, Elabscience, China), the antioxidant indices and iron level including MDA concentrations, T-SOD/CuZn-SOD activities, and Fe^2+^ concentrations were detected via colorimetry.

### Detection of FTH1 protein expression in THLE-2 cells using Western blot

The protein concentration of the samples was determined using the BCA Protein Assay Kit (Biyuntian, China). Protein samples were mixed with 5 × loading buffer and denatured at 100 °C for 5 min. Equal amounts of total protein (100 μg per lane) were separated by SDS‑PAGE with a constant voltage of 90 V for the stacking gel and 100 V for the separation gel until the target protein migrated to the lower one‑third of the separating gel for optimal resolution. Electrophoresed proteins were transferred onto PVDF membranes, which were briefly activated in methanol. The transfer was performed at 100 V and 100 mA at 4 °C. Membranes were blocked with 5% non‑fat milk in TBST for 2 h at room temperature, then incubated overnight at 4 °C with primary antibodies against GAPDH (1:5000) and FTH1 (1:1000). After washing with TBST three to four times (15 min per wash), membranes were incubated with HRP‑conjugated secondary antibody (1:1000) at 37 °C for 2 h. After extensive washing, immunoreactive bands were visualized using ECL chemiluminescent substrate and captured with a fully automatic chemiluminescence imaging system.

### Statistical analysis

An FDR cutoff of ≤ 0.01 for peptides and proteins was used as the quality control standard to screen credible identifications prior to quantitative and differential analysis. Subsequently, proteins with fold change (FC) ≥ 1.2 (upregulated) or FC ≤ 0.83 (downregulated) and *p* < 0.05 were defined as DEPs. From the ubiquitinated peptides identified in the ubiquitylome, entries with FC ≥ 2.0 (upregulated) or FC ≤ 0.50 (downregulated) and *p* < 0.05 were identified as differentially ubiquitinated peptides, and their corresponding proteins were defined as DUPs. Data for antioxidant indices, FTH1 protein expression level, and Fe^2+^ concentration were analyzed by one-way ANOVA using SAS 9.4 software. Tukey’s multiple range test was used for post-hoc pairwise comparisons between groups. Differences were considered significant at *p* < 0.05 and highly significant at *p* < 0.01.

## Supplementary Information

Additional file 1: Overview of protein identification and quantification.

Additional file 2: Overview of ubiquitinated peptide identification and quantification.

Additional file 3: Fig. S1. Domain analysis of DEPs and DUPs (top 20). Fig. S2. Expression patterns of CYP2E1 in porcine liver using TMT analysis and PRM validation. Fig. S3. Plasma antioxidant capacity and growth performance of oxidatively stressed finishing pigs (n = 6, values sharing the same letter are not significantly different (*p* > 0.05), while values without a common letter are significantly different (*p* < 0.05). Fig. S4. Total protein abundance of porcine liver in each group (A, n = 6) and total abundance of Kub protein in porcine liver in each group (B, n = 2).

Additional file 4: Table S1. Composition and nutrient levels of the experimental diet (as-fed basis) %. Table S2. Proteome identification and quantitation parameter. Table S3. Ubiquitylome identification and quantitation parameter.

Additional file 5: THLE-2 Cell STR, COA, and Mycoplasma Test Report Test.

Additional file 6: Oxidative stress cell model construction and LA concentration pre-experiment.

## Data Availability

Data will be made available on request. The raw mass spectrometry data have been deposited in the iProX database (ProteomeXchange Consortium) with accession number IPX0018599000.

## Abbreviations

ACE: Acevaltrate
ACSL4: Acyl-CoA synthetase long-chain family member 4
ADFI: Average daily feed intake
ADG: Average daily weight gain
ALDH2: Aldehyde dehydrogenase 2
BP: Biological process
CAT: Catalase
CC: Cellular component
CK: Control group
Co-IP: Co‑immunoprecipitation
CPR: Cytochrome P450 Reductase
CuZn-SOD: Copper‑zinc superoxide dismutase
DEPs: Differentially expressed proteins
DHLA: Dihydrolipoic acid
DL: Diquat + LA group
DQ: Diquat/Diquat group
DTT: Dithiothreitol
DUBs: Deubiquitinases
DUPs: Differentially ubiquitinated proteins
FASP: Filter-aided sample preparation
FC: Fold change
FTH1: Ferritin heavy chain 1
FTL: Ferritin light chain
GCL: Glutamate cysteine ligase
GPX4: Glutathione peroxidase 4
GSH: Glutathione
GSH/GSSG: Glutathione (Reduced/Oxidized)
GSH-Px: Glutathione peroxidase
GSS: Glutathione synthase
H_2_O_2_: Hydrogen peroxide
HE: Hematoxylin and eosin
IAA: Iodoacetamide
IAP: Immunoaffinity purification
IB: Immunoblotting
IP: Immunoprecipitation
Keap1: Kelch-like ECH-associated protein 1
KEGG: Kyoto Encyclopedia of Genes and Genomes
LA: α-Lipoic acid
LC–MS/MS: Liquid chromatography-tandem mass spectrometry
LW: Large White
MDA: Malondialdehyde
MF: Molecular function
4-HNE: 4-Hydroxynonenal
NADP^+^/NADPH: Nicotinamide adenine dinucleotide phosphate (Oxidized/Reduced)
NCOA4: Nuclear receptor coactivator 4
${\mathrm{O}}_{2}^{\cdot -}$: Superoxide anion radical
PCBP1: Poly(rC)-binding protein 1
PRM: Parallel reaction monitoring
PTMs: Post-translational modifications
ROS: Reactive oxygen species
SLC3A2: Solute carrier family 3 member 2
SLC7A11: Solute carrier family 7 member 11
SOD: Superoxide dismutase
System Xc^−^: Cystine/glutamate antiporter system
T-AOC: Total antioxidant capacity
TFA: Trifluoroacetic acid
TfR1: Transferrin receptor 1
THLE-2: Transformed human liver epithelial-2
TMT: Tandem mass tag
T-SOD: Total superoxide dismutase
