# Supplementary material for: Ubiquitylome analysis reveals the protective effect of α-lipoic acid via the ferroptosis pathway during recovery after hepatic oxidative stress in finishing pigs
Source: Stress Biol. 2026 Sep 23;6(1):68. doi: 10.1007/s44154-026-00341-1 (PMC13601454; doi:10.1007/s44154-026-00341-1)
Supplement: Supplementary file 1 [file 44154_2026_341_MOESM3_ESM.pdf]

Additional file 3

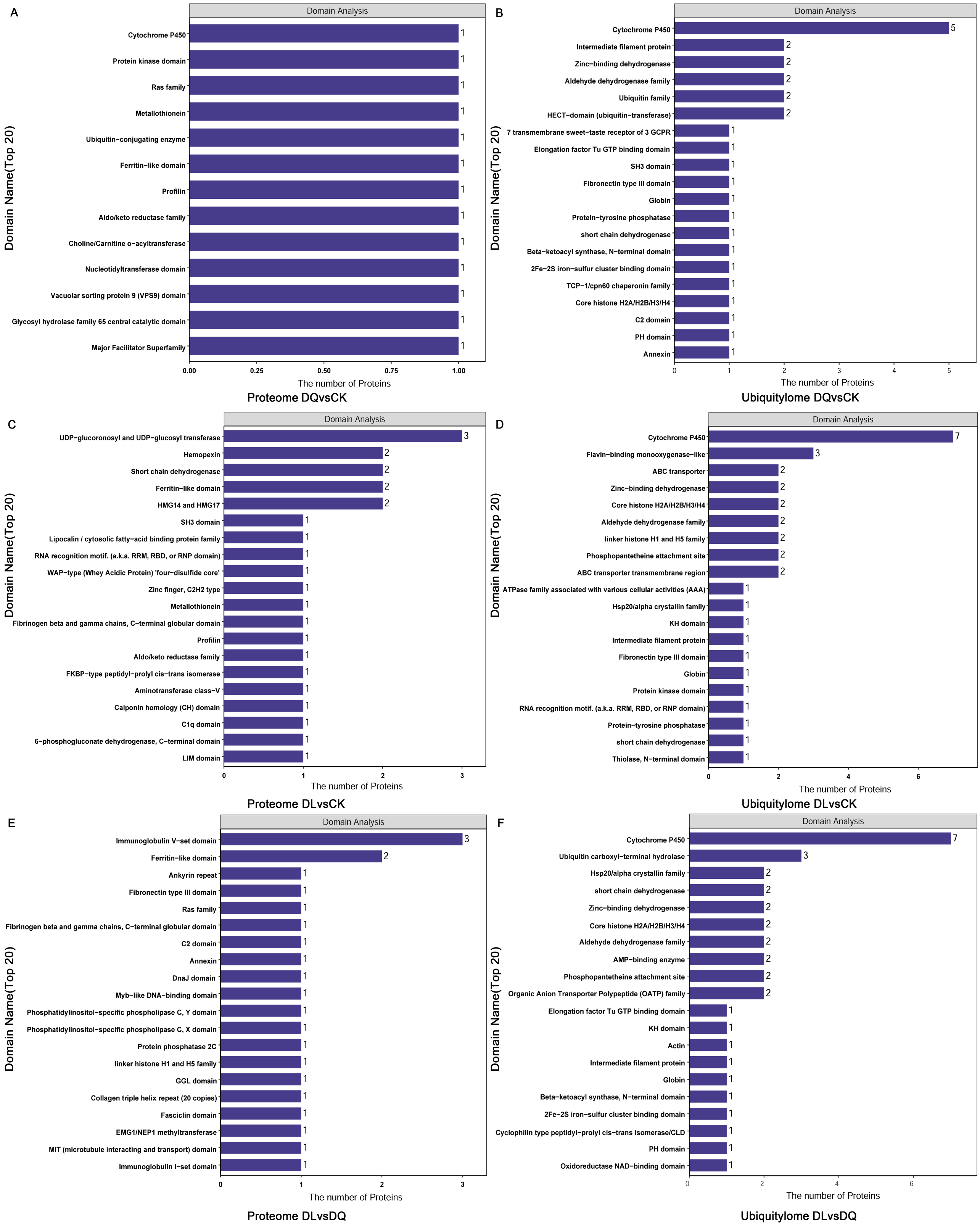

Note: Display the top 20 entries in ascending order of *p-value*, or all entries if fewer than 20.

Fig. S1 Domain analysis of DEPs and DUPs (top 20)

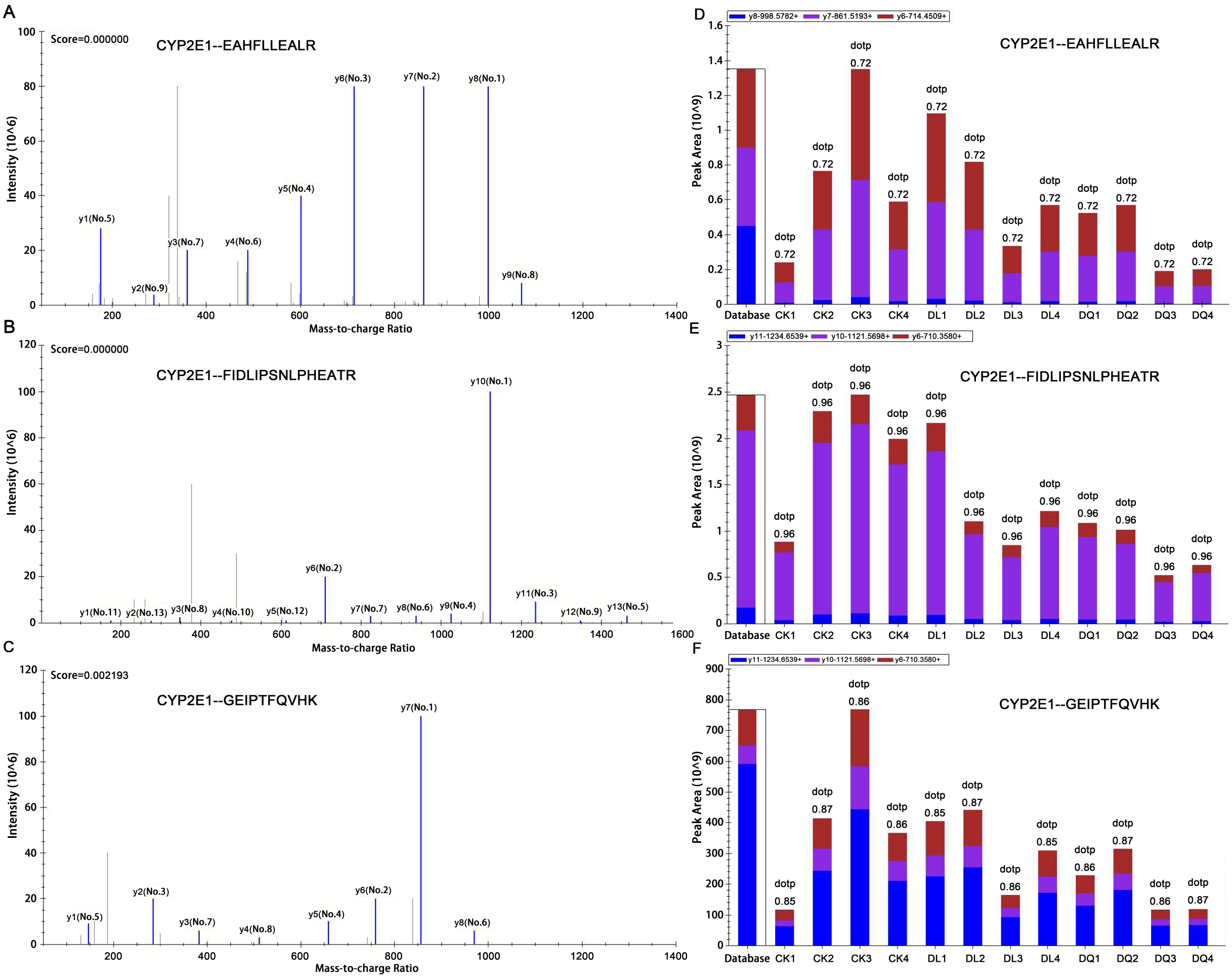

Note: (A-C) the secondary mass spectrum of candidate peptide fragments of the CYP2E1 protein is shown as an example; (D-F) the bar chart compares the peak areas of three specific peptide fragments of the CYP2E1 protein, with three different colors representing the three sub-ion peaks; n=4.

Fig. S2 Expression patterns of CYP2E1 in porcine liver using TMT analysis and PRM validation

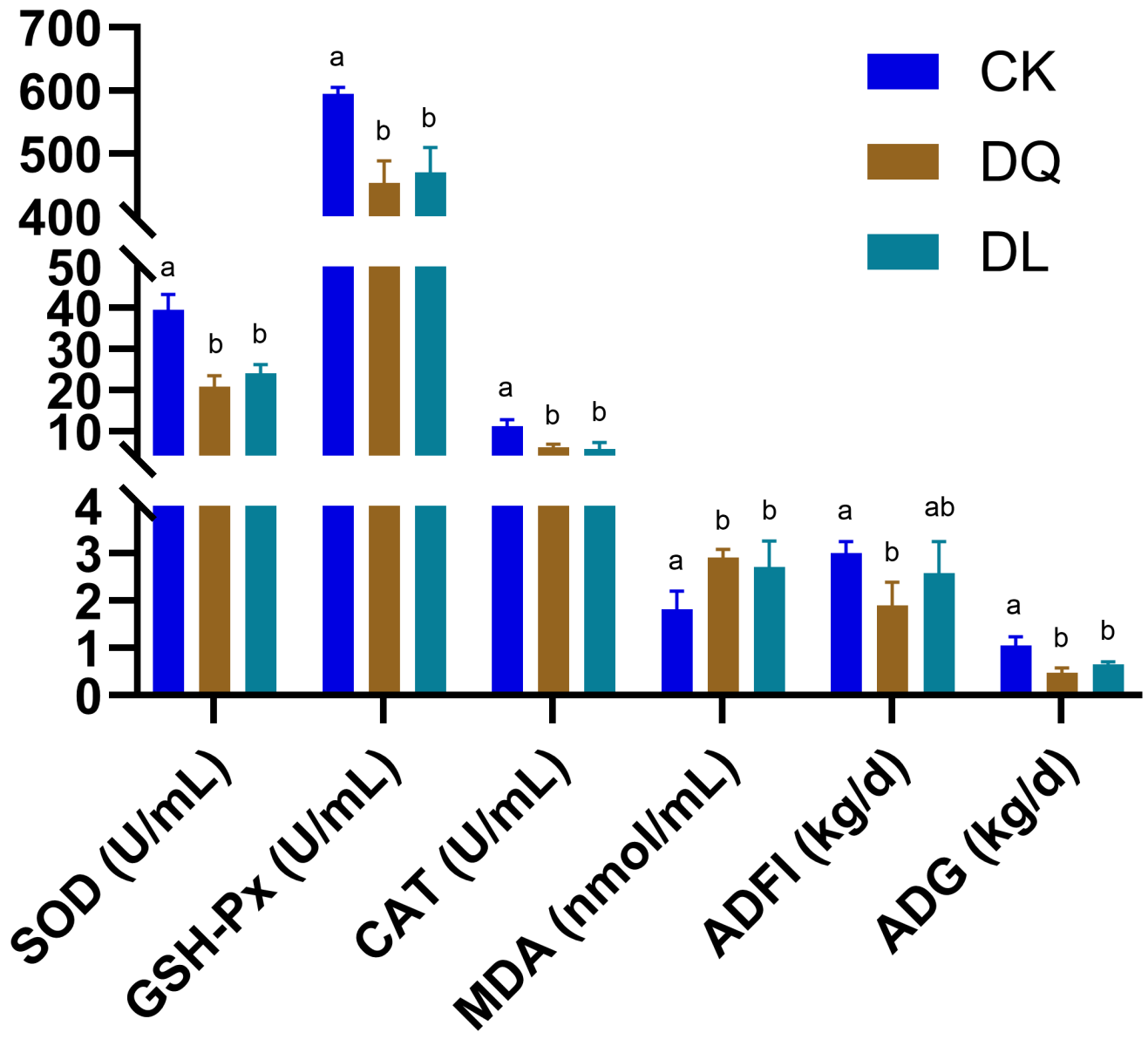

Fig. S3 Plasma antioxidant capacity and growth performance of oxidatively stressed finishing pigs (n = 6, values sharing the same letter are not significantly different ($P > 0.05$), while values without a common letter are significantly different ($P < 0.05$).

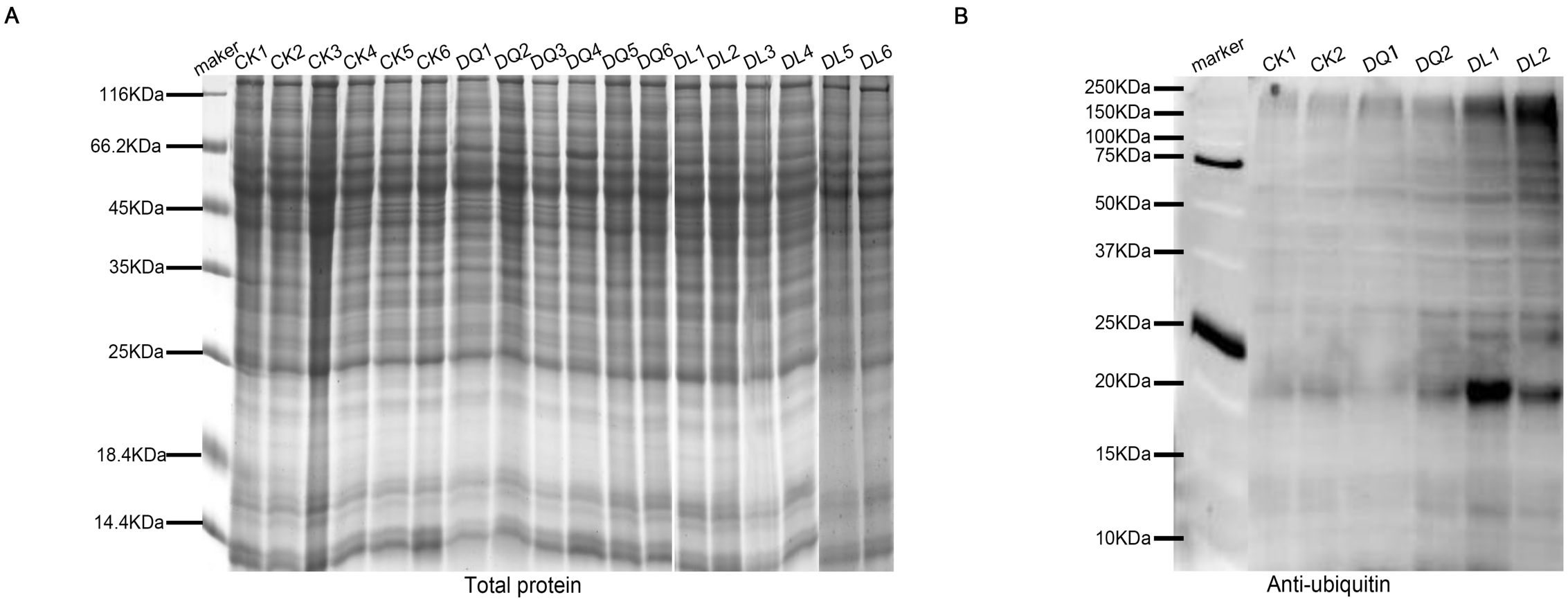

Fig. S4 Total protein abundance of porcine liver in each group (A, n = 6) and total abundance of Kub protein in porcine liver in each group (B, n = 2)
