# Supplementary material for: Ubiquitylome analysis reveals the protective effect of α-lipoic acid via the ferroptosis pathway during recovery after hepatic oxidative stress in finishing pigs
Source: Stress Biol. 2026 Sep 23;6(1):68. doi: 10.1007/s44154-026-00341-1 (PMC13601454; doi:10.1007/s44154-026-00341-1)
Supplement: Supplementary file 2 [file 44154_2026_341_MOESM4_ESM.pdf]

Additional file 4

Table S1 Composition and nutrient levels of the experimental diet (as-fed basis) %.

Items	Content
Ingredients	
Corn	66.20
Soybean meal (42.8% CP)	20.00
Wheat bran	6.50
Wheat middlings	4.00
Limestone	1.00
$CaHPO_4$	0.60
NaCl	0.40
Premix[1)]	1.00
L-Lys·HCl	0.30
Total	100.00
Nutrient levels[2)]	
Digestible energy (MJ/kg)	13.39
Crude protein (%)	15.73
Calcium (%)	0.65
Total phosphorus (%)	0.41
Available phosphorus (%)	0.17
Lysine (%)	0.92
Met+Cys (%)	0.54

[1)] Premix provided the following per kg of the diet: vitamin A 2512 IU, vitamin D_3 1200 IU, vitamin E_3 4 IU, vitamin K_3 1.5 mg, vitamin B_{12} 17.6 μg, riboflavin 2.5 mg, pantothenic acid 6.8 mg, nicotinic acid 20.3 mg, choline 351 mg, Mn 10 mg, Fe 50 mg, Zn 50 mg, Cu 10 mg, I 0.3 mg, Se 0.3 mg. [2)] Digestible energy (DE) and available phosphorus (AP) were calculated values, while the others were measured values.

Table S2 Proteome identification and quantitation parameter.

Item	Value
Enzyme	Trypsin
Max Missed Cleavages	2
Peptide Mass Tolerance	± 20 ppm
Fragment Mass Tolerance	0.1Da
Fixed modifications	Carbamidomethyl (C)
	TMT 6/10/16 plex (N-term), TMT 6/10/16 plex (K)
Variable modifications	Oxidation (M)
Database	uniprot_Sus_scrofa_122045_20210125.fasta
Database pattern	Decoy
Peptide FDR	≤ 0.01
Protein Quantification	The protein ratios are calculated as the median of only unique peptides of the protein.
Experimental Bias	Normalizes all peptide ratios by the median protein ratio. The median protein ratio should be 1 after the normalization.

Note: FDR = false discovery rate.

Table S3 Ubiquitylome identification and quantitation parameter.

Item	Value
Enzyme	Trypsin
Max Missed Cleavages	2
Main search	6 ppm
First search	20 ppm
MS/MS Tolerance	20 ppm
Fixed modifications	Carbamidomethyl (C)
Variable modifications	Oxidation (M) , Acetyl (Protein N-term), GlyGly (K)
Database	uniprot_Sus_scrofa_122045_20210125.fasta
Database pattern	Reverse
Include contaminants	True
Site FDR	≤ 0.01
Protein FDR	≤ 0.01
Time window (match between runs)	2 min

Note: FDR = false discovery rate.
