# Supplementary material for: Ubiquitylome analysis reveals the protective effect of α-lipoic acid via the ferroptosis pathway during recovery after hepatic oxidative stress in finishing pigs
Source: Stress Biol. 2026 Sep 23;6(1):68. doi: 10.1007/s44154-026-00341-1 (PMC13601454; doi:10.1007/s44154-026-00341-1)
Supplement: Supplementary file 3 [file 44154_2026_341_MOESM5_ESM.pdf]

GENETIC TESTING BIOTECHNOLOGY

Cell Line Authentication Service
STR Profile Report

**Sample Submitted By:**	Dr. Zuokang Zheng<br>Zhejiang Meisen Cell Technology Co.,Ltd.
**Email Address:**	947638289@qq.com wzppanda@163.com
**Sales Order:**	220715K
**Cell Line Designation:**	THLE-2
**Date Sample Received:**	Jul 15th, 2022
**Report Date:**	Jul 18th, 2022

Methodology: Twenty-one short tandem repeat (STR) loci, one Y-Indel locus plus the Amelogenin locus were amplified using the commercially available SiFaSTR™ 23 plex Kit. The cell line sample was processed using the ABI Prism® 3130 XL Genetic Analyzer. Data were analyzed using GeneMapper® ID v3.2 software (Applied Biosystems). Appropriate positive and negative controls were run and confirmed for each sample submitted.

Data Interpretation: Cell lines were authenticated using Short Tandem Repeat (STR) analysis as described in 2012 in ANSI Standard (ASN-0002) by the ATCC Standards Development Organization (SDO) and in Capes-Davis et al., Match criteria for human cell line authentication: Where do we draw the line? Int J Cancer. 2013;132(11):2510-9.

GTB™ performs STR Profiling following ISO 9001:2008 and ISO/IEC 17025:2005 quality standards.

There are no warranties with respect to the services or results supplied, express or implied, including, without limitation, any implied warranty of merchantability or fitness for a particular purpose. Genetic Testing Biotechnology (GTB) is not liable for any damages or injuries resulting from receipt and/or improper, inappropriate, negligent or other wrongful use of the test results supplied, and/or from misidentification, misrepresentation, or lack of accuracy of those results. Your exclusive remedy against GTB and those supplying materials used in the services for any losses or damage of any kind whatsoever, whether in contract, tort, or otherwise, shall be, at GTB's option, refund of the fee paid for such service or repeat of the service.

NOTE: According to the recommendations of *IJC* on cell line authentication, the report is valid for 3 years since the issue date.

Technical Questions?
GTB Technical Support
+86-512-67486171
service@jsdna.org
Section 505, Yixin BLD
SIP, Suzhou, 215123
Jiangsu, P.R. China

Ordering Questions?
order@jsdna.org
GTB Corporation
+86-512-62806339
Section 303, Yixin BLD
SIP, Suzhou, 215123
Jiangsu, P.R. China

Cell Line Authentication Service
STR Profile Report

Sales Order: 220715K

	Test Results for Submitted Sample		ExPASy Reference Database Profile	
**Loci**	**Query Profile:** THLE-2		**Database Profile:** THLE-2	
Amelogenin	X	Y	X	Y
D3S1358	14	15		
D5S818	11	13	11	13
D2S1338	17	23		
TPOX	8	11	8	11
CSF1PO	11	13	11	13
Penta D	10	13		
Y- Indel	2			
TH01	7	9.3	7	9.3
vWA	16	17	16	17
D7S820	10	12	10	12
D21S11	30	32.2		
Penta E	12	13		
D10S1248	13	14		
D8S1179	13			
D1S1656	11	17		
D18S51	13	16		
D12S391	21	23		
D6S1043	12	18		
D19S433	14			
D16S539	11	13	11	13
D13S317	8	12	8	12
FGA	22	24		

The allele match algorithm compares the 8 core loci plus amelogenin only, even though alleles from all loci will be reported when available.

Note: Loci highlighted in grey (8 core STR loci plus Amelogenin) can be made public to verify cell identity. In order to protect the identity of the donor, ***please do not publish*** *the allele calls from all the STR loci tested.*

The sample match is based on the reference data available at the time of comparison.

Explanation of Test Results

Cell lines with ≥80% match are considered to be related; i.e., derived from a common ancestry. Cell lines with between a 55% to 80% match require further profiling for authentication of relatedness.

- ☐ The submitted sample profile is human, but not a match for any profile in the ExPASy STR database.
- ☑ The submitted profile is an exact match for the following human cell line(s) in the ExPASy STR database (8 core loci plus Amelogenin): THLE-2
- ☐ The submitted profile is similar to the following ExPASy human cell line(s):

e-Signature Technician: ____________________

e-Signature Reviewer: ____________________

More information

GENETIC TESTING BIOTECHNOLOGY

Cell Line Authentication Service
STR Profile Report

Addendum: Electropherogram for the customer's sample set 1 of 1

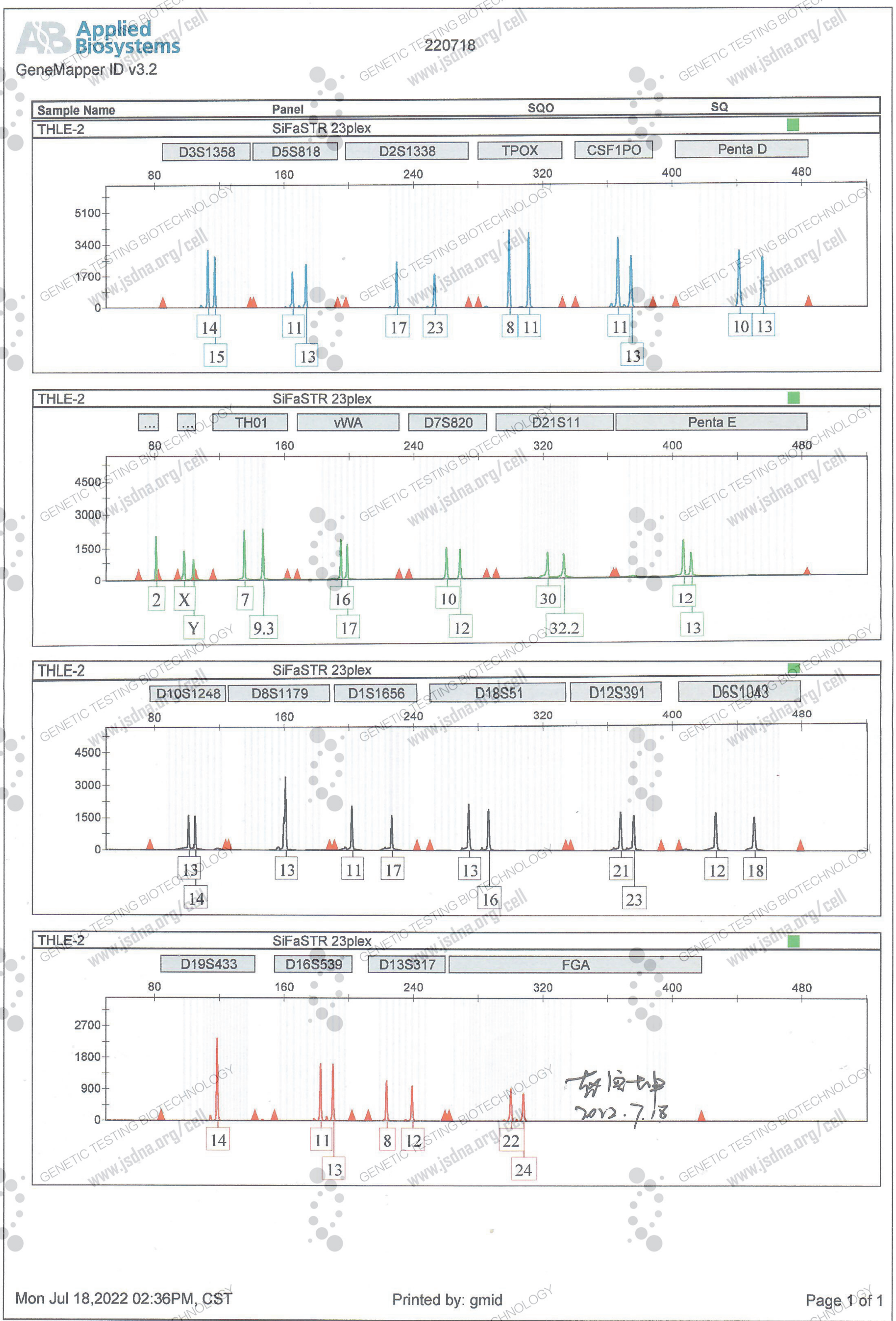

Accession	Name	N° Markers	Score	Amel	CSF1PO	D2S1338	D3S1358	D5S818	D7S820	D8S1179	D13S317	D16S539	D18S51	D19S433	D21S11	FGA	Penta D	Penta E	TH01	TPOX	vWA
NA	Query	NA	NA	X,Y	11,13			11,13	10,12		8,12	11,13							7,9.3	8,11	16,17
CVCL_3803	THLE-2	9	100.00%	X,Y	11,13			11,13	10,12		8,12	11,13							7,9.3	8,11	16,17
CVCL_VP83	ESi063-A	9	80.00%	X,Y	11,13			11	10,12		11,12	11,13			28,29				6,7	8,11	17,19
CVCL_6903	GBC-SD	9	78.79%	X,Y	13	17,24	17	11	11,12	10,12	8,12	11,13	14,21	14,15.2	30,31	23			7,10	11	16,17
CVCL_A9DL	GBC-SD-Cas9-766	9	78.79%	X,Y	11	17,24	17	11	11,12	10,12	8,12	11,13	14,21	14,15.2	30,31	23		12	7,10	11	16,17
CVCL_A9DM	GBC-SD-Cas9-767	9	78.79%	X,Y	11	17,24	17	11	11,12	10,12	8,12	11,13	14,21	14,15.2	30,31	23		12	7,10	11	16,17
CVCL_A9DN	GBC-SD-Cas9-768	9	78.79%	X,Y	11	17,24	17	11	11,12	10,12	8,12	11,13	14,21	14,15.2	30,31	23		12	7,10	11	16,17
CVCL_A9DP	GBC-SD-Cas9-769	9	78.79%	X,Y	11	17,24	17	11	11,12	10,12	8,12	11,13	14,21	14,15.2	30,31	23		12	7,10	11	16,17
CVCL_A9DQ	GBC-SD-Cas9-770	9	78.79%	X,Y	11	17,24	17	11	11,12	10,12	8,12	11,13	14,21	14,15.2	30,31	23		12	7,10	11	16,17
CVCL_A9DR	GBC-SD-Cas9-771	9	78.79%	X,Y	11	17,24	17	11	11,12	10,12	8,12	11,13	14,21	14,15.2	30,31	23		12	7,10	11	16,17
CVCL_A9DS	GBC-SD-Cas9-772	9	78.79%	X,Y	11	17,24	17	11	11,12	10,12	8,12	11,13	14,21	14,15.2	30,31	23		12	7,10	11	16,17
CVCL_A9DT	GBC-SD-Cas9-773	9	78.79%	X,Y	11	17,24	17	11	11,12	10,12	8,12	11,13	14,21	14,15.2	30,31	23		12	7,10	11	16,17
CVCL_A9DU	GBC-SD-Cas9-774	9	78.79%	X,Y	11	17,24	17	11	11,12	10,12	8,12	11,13	14,21	14,15.2	30,31	23		12	7,10	11	16,17
CVCL_A9DV	GBC-SD-Cas9-775	9	78.79%	X,Y	11	17,24	17	11	11,12	10,12	8,12	11,13	14,21	14,15.2	30,31	23		12	7,10	11	16,17
CVCL_A9DK	GBC-SD-Luc2-tdT	9	78.79%	X,Y	11	17,24	17	11	11,12	10,12	8,12	11,13	14,21	14,15.2	30,31	23		12	7,10	11	16,17
CVCL_RP28	MESO-27T	9	78.79%	X,Y	11,13		15,17	11,13	10,11	8,13	12,13	13	13		29,30	23	9	7,10	9.3	8	16,17
CVCL_2647	NCI-BL2052	9	78.79%	X,Y	10,11			11	11,12		12	11,13							7,9.3	8,11	17
CVCL_XE03	ZJU-0430	9	78.79%	X,Y	11	17,24	17	11	11,12	10,12	8,12	11,13	14,21	14,15.2	30,31	23	11,12	12	7,10	11	16,17
CVCL_1518 *Best*	NCI-H2052	9	77.42%	X	10,11		14,15	11	12	13	12	11,13	17		32.2	24	10	12,16	7,9.3	8,11	17

Meisen CTCC

CERTIFICATE OF ANALYSIS

CTCC Numer: CTCC-004-0030
Lot Number: M21SJ1304

Name: THLE-2
Description: Liver
Species: Human(*Homo sapiens*)
Volume/Ampule: Approximately 1mL
Date Frozen: 04 Feb. 2021
Recovery: A T-25 setup at a seeding density of 5×10^4 viable cells/cm^2 reaches approximately 80% confluence in 4 days.
Product Format: Cells cryopreserved in the appropriate cryropreservation medium
Expiration Date: Not applicable
Storage Conditions: Vapor phase of liquid nitrogen

Test / Method	Specification	Result
**Ampule passage number**	Report results	1
**Population doubling level (PDL)**	Report results	Not applicable
**Total cells / ampule** (Cell counting using Trypan Blue stain method)	Report results	2×10^6 total cells/ampule
**Post-freeze viability** (Cell counting using Trypan Blue stain method)	⩾50.0%	92%
**Growth properties** (Visual observation method)	Adherent	Adherent
**Morphology** (Visual observation method)	Epithelial-like	Epithelial-like
**Test for mycoplasma contamination** (PCR-based assay)	None detected	None detected
**Human pathogenic virus testing** (PCR-based assay for HIV, HepB, HPV, EBV, and CMV)	Report results	HIV - None detected<br>HepB - None detected<br>HPV - None detected<br>EBV - None detected<br>CMV - None detected

China Tissue Culture Collection
Panan, Zhejiang Province,
P.R China
www.ctcc.online

Tel: 0571-86027729

Eail: meisencell@ctcc.online

CERTIFICATE OF ANALYSIS

CTCC Numer: CTCC-004-0030
Lot Number: M23SJ1304

Test / Method

Species determination: STR analysis (intraspecies)

	Test Results for Submitted Sample		ExPASy Reference Database Profile	
Loci	Query Profile: THLE-2		Database Profile: THLE-2	
Amelogenin	X	Y	X	Y
D3S1358	14	15		
D5S818	11	13	11	13
D2S1338	17	23		
TPOX	8	11	8	11
CSF1PO	11	13	11	13
Penta D	10	13		
Y- Indel	2			
TH01	7	9.3	7	9.3
vWA	16	17	16	17
D7S820	10	12	10	12
D21S11	30	32.2		
Penta E	12	13		
D10S1248	13	14		
D8S1179	13			
D1S1656	11	17		
D18S51	13	16		
D12S391	21	23		
D6S1043	12	18		
D19S433	14			
D16S539	11	13	11	13
D13S317	8	12	8	12
FGA	22	24		

Signed by Jim Kang

Date: 2021.02.09

Quality Assurance Specialist; Quality Assurance

CTCC hereby represents and warrants that the material provided under this certificate is pure and has been subjected to the tests and procedures specified and that the results described, along with any other data provided in this certificate, are true and correct to the best of the companyis knowledge and belief. This certificate does not extend to the growth and/or passage of any living organism or cell line beyond what is supplied within the container received from CTCC.

This product is intended to be used for laboratory research use only. It is not intended for use in humans, animals,or for diagnostics. Appropriate Biosafety Level (BSL) practices should always be used with this material. Refer to the Product Information Sheet for instructions on the correct use of this product.

CTCC products may not be resold, modified for resale, used to provide commercial services, or to manufacture commercial products without prior written agreement from CTCC.

China Tissue Culture Collection
Panan, Zhejiang Province,
P.R China
www.ctcc.online

Tel: 0571-86027729

Eail: meisencell@ctcc.online

Mycoplasma Test Report Test

Date: Sep 28, 2022 1.

Test Samples Code (-): Negative control

Code (+): Positive control

Code (1): THLE-2 cell culture supernatant (cultured for 72 h)

2. Test Method & Results

Mycoplasma detection was performed via PCR using a commercial mycoplasma detection kit (HUABIO, Cat# K0103) following the manufacturer's standard protocol. Gel electrophoresis image of PCR products is attached for reference.

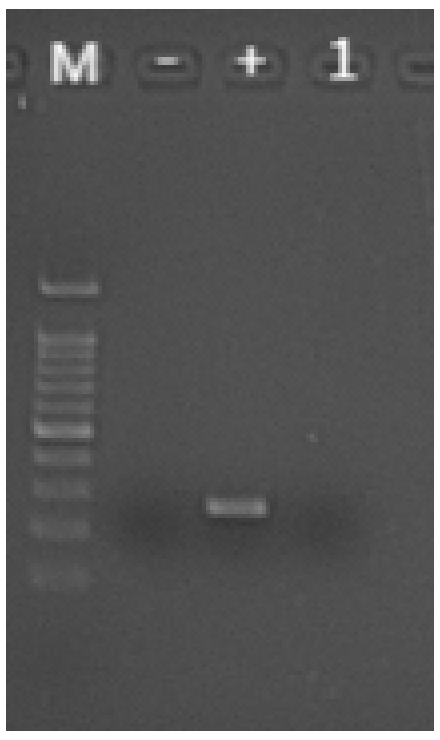

3. Conclusion No mycoplasma contamination was detected in the tested THLE-2 cell supernatant sample.
