# Supplementary material for: Ubiquitylome analysis reveals the protective effect of α-lipoic acid via the ferroptosis pathway during recovery after hepatic oxidative stress in finishing pigs
Source: Stress Biol. 2026 Sep 23;6(1):68. doi: 10.1007/s44154-026-00341-1 (PMC13601454; doi:10.1007/s44154-026-00341-1)
Supplement: Supplementary file 4 [file 44154_2026_341_MOESM6_ESM.pdf]

Additional file 4

Oxidative stress cell model construction and LA concentration pre-experiment

① Under a fixed treatment duration of 24 h, THLE-2 cells were treated with gradient LA concentrations (0, 25, 50, 100, 200, 400, 800 μM). The results demonstrated that 200, 400 and 800 μM LA significantly improved cell viability (P < 0.01, Fig. S5).

② With a fixed treatment duration of 12 h, hepatocytes were challenged with 50, 100 and 200 μM H_2O_2. The concentration of 100 μM H_2O_2 markedly reduced cell viability (P < 0.001) and CuZn-SOD activity (P < 0.01), while significantly elevated the level of MDA (P < 0.01), which was identified as the optimal concentration for oxidative stress modeling (Fig. S6).

③ Under the fixed H_2O_2 concentration of 100 μM, cells were treated for 6, 12, 24 and 48 h. The 24 h treatment led to significant decreases in cell viability (P < 0.01), total SOD (T-SOD, P < 0.05) and CuZn-SOD activity (P < 0.01), resulting in stable oxidative damage (Fig. S7).

In summary, we set the formal in vitro experimental conditions as follows: THLE-2 hepatocytes were pretreated with three graded LA concentrations (200, 400 and 800 μM) for 24 h, and subsequently challenged with 100 μM H_2O_2 for another 24 h.

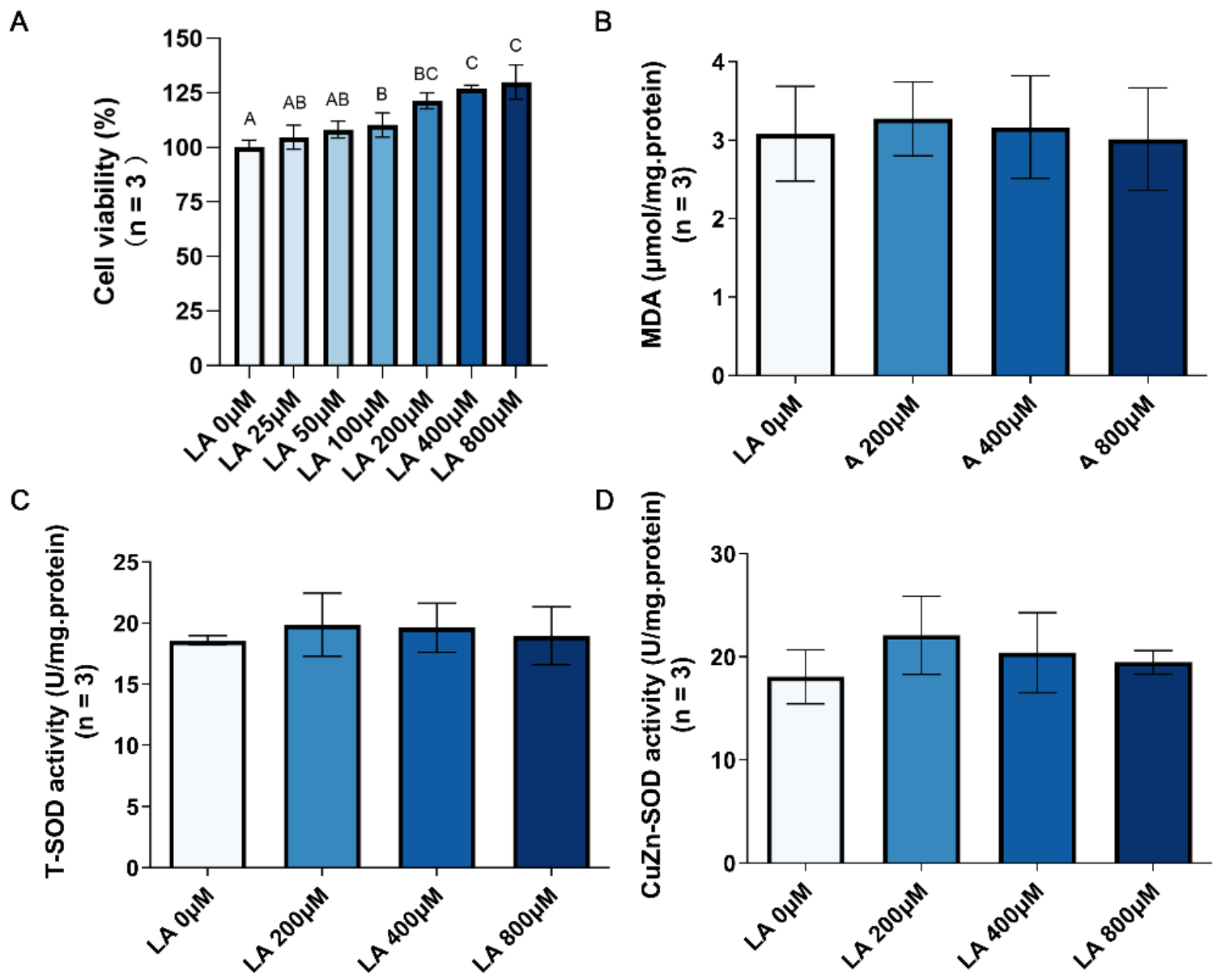

Note: Effects of different LA concentrations on (A) cell viability, (B) MDA content, (C) T-SOD activity and (D) CuZn-SOD activity in THLE-2 cells. Values with different letters indicate significant differences between two groups (P < 0.05); otherwise, there is no significant difference.

Fig. S5 Effects of α-lipoic acid (LA) on cell viability, antioxidant level in THLE-2 cell

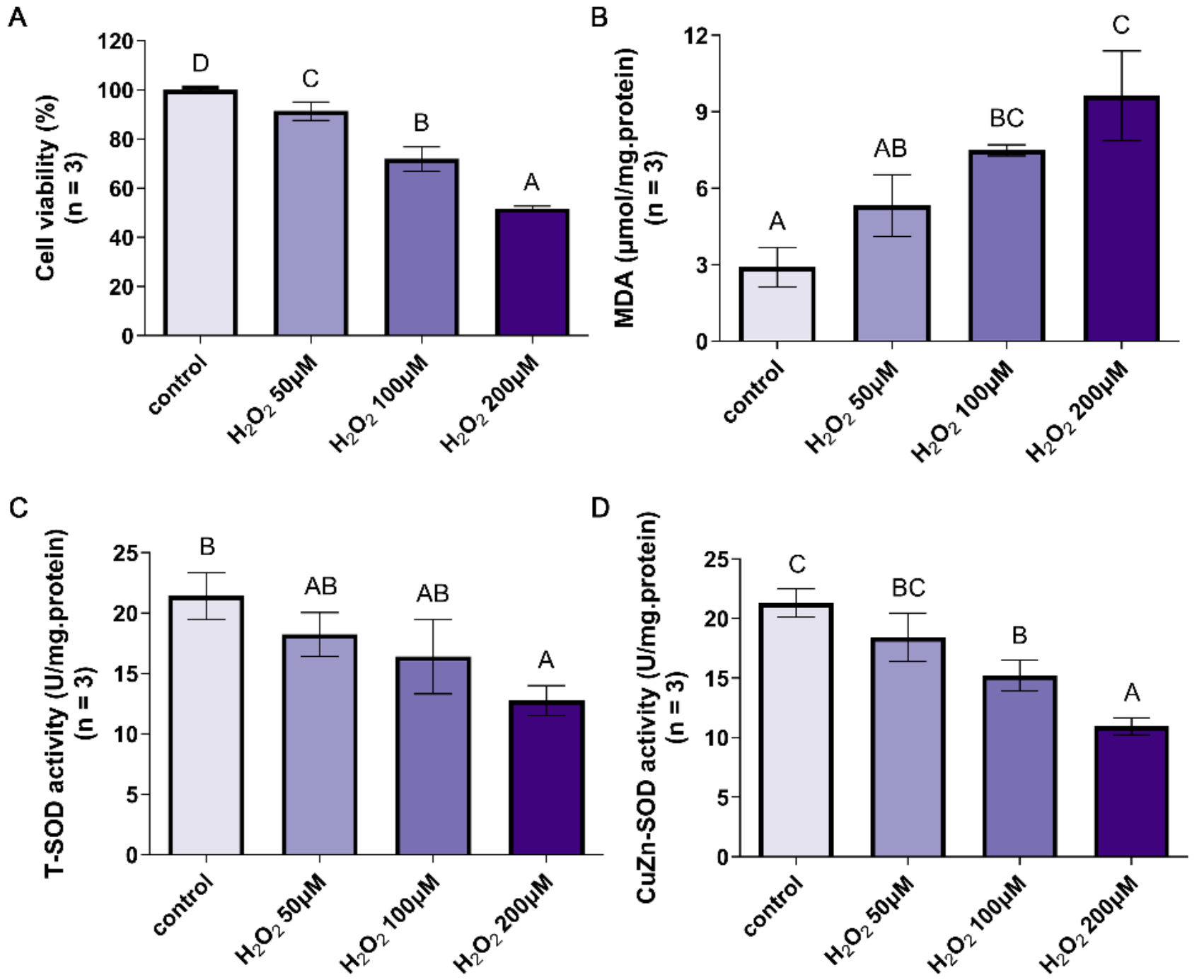

Note: Effects of 12 h treatment with different concentrations of H_2O_2 on (A) cell viability, (B) MDA content, (C) T-SOD activity and (D) CuZn-SOD activity in THLE-2 cells. Values with different letters indicate significant differences between two groups ($P < 0.05$); otherwise, there is no significant difference.

Fig. S6 Effective concentration of H_2O_2 for inducing oxidative stress in THLE-2 cell

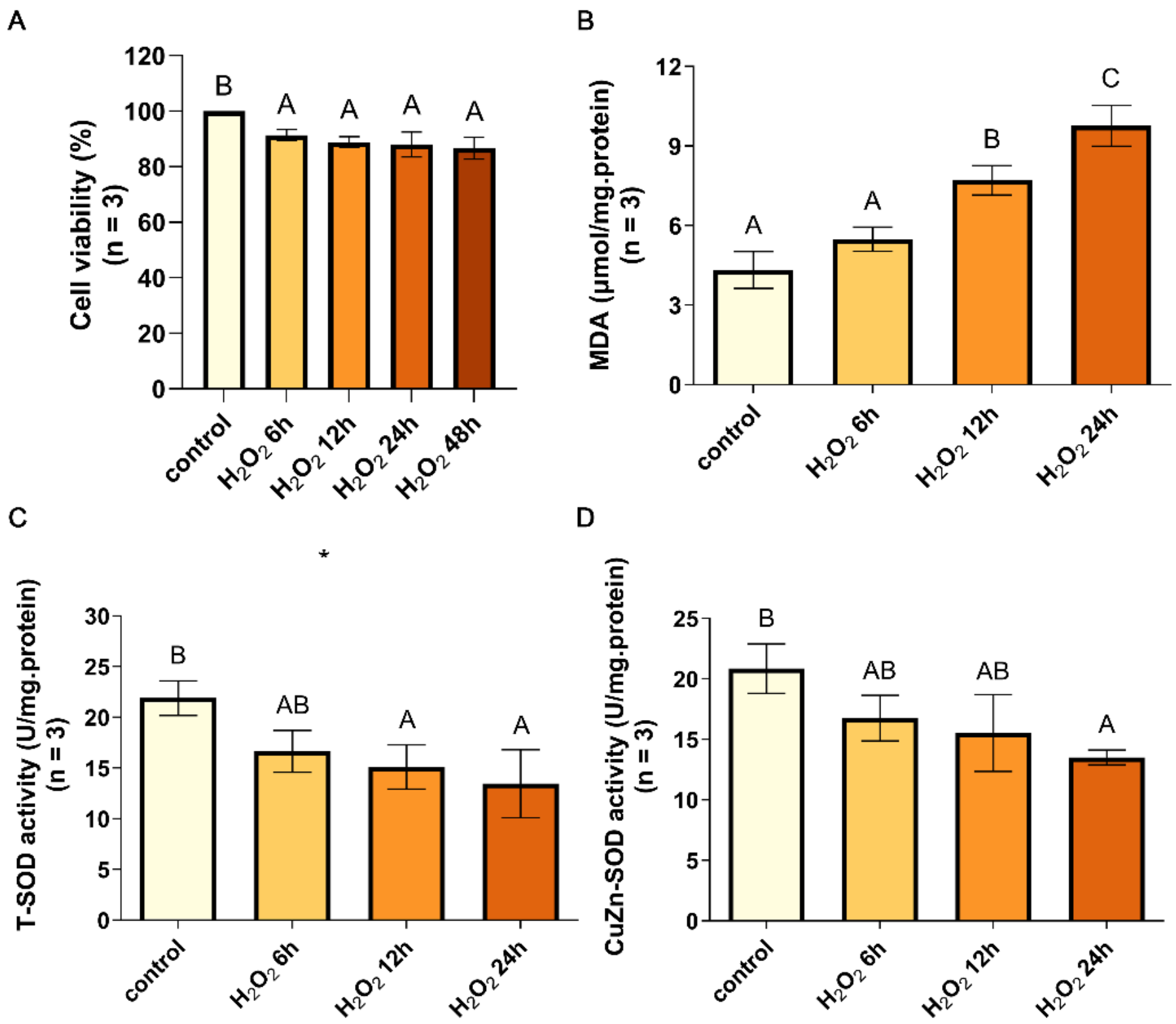

Note: Effects of different treatment durations with 100 μM H_2O_2 on (A) cell viability, (B) MDA content, (C) T-SOD activity and (D) CuZn-SOD activity in THLE-2 cells. Values with different letters indicate significant differences between two groups ($P < 0.05$); otherwise, there is no significant difference.

Fig. S7 Effective duration of H_2O_2 for inducing oxidative stress in THLE-2 cell
